# Hybrid Sterility, Genetic Conflict and Complex Speciation: Lessons From the *Drosophila simulans* Clade Species

**DOI:** 10.3389/fgene.2021.669045

**Published:** 2021-06-23

**Authors:** Daven C. Presgraves, Colin D. Meiklejohn

**Affiliations:** ^1^Department of Biology, University of Rochester, Rochester, NY, United States; ^2^School of Biological Sciences, University of Nebraska, Lincoln, NE, United States

**Keywords:** speciation, Haldane’s rule, large X-effect, genetic conflict, gene flow

## Abstract

The three fruitfly species of the *Drosophila simulans* clade— *D. simulans, D. mauritiana*, and *D. sechellia*— have served as important models in speciation genetics for over 40 years. These species are reproductively isolated by geography, ecology, sexual signals, postmating-prezygotic interactions, and postzygotic genetic incompatibilities. All pairwise crosses between these species conform to Haldane’s rule, producing fertile F_1_ hybrid females and sterile F_1_ hybrid males. The close phylogenetic proximity of the *D. simulans* clade species to the model organism, *D. melanogaster*, has empowered genetic analyses of their species differences, including reproductive incompatibilities. But perhaps no phenotype has been subject to more continuous and intensive genetic scrutiny than hybrid male sterility. Here we review the history, progress, and current state of our understanding of hybrid male sterility among the *D. simulans* clade species. Our aim is to integrate the available information from experimental and population genetics analyses bearing on the causes and consequences of hybrid male sterility. We highlight numerous conclusions that have emerged as well as issues that remain unresolved. We focus on the special role of sex chromosomes, the fine-scale genetic architecture of hybrid male sterility, and the history of gene flow between species. The biggest surprises to emerge from this work are that (*i*) genetic conflicts may be an important general force in the evolution of hybrid incompatibility, (*ii*) hybrid male sterility is polygenic with contributions of complex epistasis, and (*iii*) speciation, even among these geographically allopatric taxa, has involved the interplay of gene flow, negative selection, and positive selection. These three conclusions are marked departures from the classical views of speciation that emerged from the modern evolutionary synthesis.

## Introduction

From Darwin’s *Origin of Species*, to the modern evolutionary synthesis (Dobzhansky, 1937), and into the present era (Coyne and Orr, 2004), hybrid incompatibility— the intrinsic sterility or inviability of species hybrids— has held a central place in speciation research. The reason, most simply, is that hybrid incompatibility has contributed to reproductive isolation during the speciation histories of some species pairs, limiting gene flow and, on occasion, spurring the evolution of further reproductive isolation via reinforcement (Dobzhansky, 1940; Yukilevich, 2012). Conventional wisdom has it, however, that, during the time-course of speciation, hybrid incompatibility evolves late or, worse, after the fact (Mallet, 2006; Rabosky and Matute, 2013). Why then bother to study hybrid incompatibility? Apart from its sometimes role in speciation, there are at least two further reasons. One is that hybrid incompatibility is the manifestation of extreme species differences in genome function, structure, content, and regulation in which *wildtype* alleles from one species *kill or sterilize* when in the genomes of closely related species. Determining the molecular identities and forces involved in the evolution of these extreme species differences is therefore informative about the most rapidly evolving aspects of essential biological functions. The other reason is that hybrid incompatibility has presented a series of puzzles for evolutionary biology. For instance: as dead or sterile hybrid progeny are of no adaptive value, how could natural selection possibly explain the evolution of hybrid incompatibility? Darwin’s (1859) solution is that hybrid incompatibility is not itself adaptive but rather “incidental on other acquired differences” (p. 245). And: given Mendelian inheritance, how is the evolution of hybrid incompatibility permissible by natural selection at all? If heterozygous (*Aa*) hybrids are sterile because *A* and *a* alleles are incompatible, then the critical substitution (*AA* → *Aa* → *aa*) would be precluded by the sterile intermediate genotype. Bateson’s (1909) solution is that hybrid incompatibility can evolve readily, without passing through problematic intermediate genotypes, so long as substitutions occur at different interacting loci [*AABB* → *aaBB* → *aabb*, with, *e.g*., the *A* and *b* alleles being incompatible; see Orr (1996)]. The Darwin and Bateson solutions were later rediscovered and deepened by the modern synthesis thinkers (Dobzhansky, 1937; Muller, 1940, 1942) and today represent starting points for most genetic studies of hybrid incompatibility. As we explain below, modern genetic analyses have left us with several new puzzles concerning the evolution, biology, and consequences of hybrid incompatibilities.

Genetic incompatibility thinking was implicit in Sturtevant’s (1920) pioneering investigations of lethality in hybrids between *Drosophila melanogaster* and the newly discovered *Drosophila simulans*. Over the decades since, crosses between these two species have leveraged the ever-expanding genetic, molecular, and genomic resources of *D. melanogaster* to reveal, among other things, that F_1_ hybrid lethality involves sex chromosomes, autosomes, maternal factors (Sturtevant, 1920), protein-coding genes (Barbash et al., 2003; Brideau et al., 2006; Phadnis et al., 2015), and repetitive DNAs (Sawamura and Yamamoto, 1997; Ferree and Barbash, 2009, Satyaki et al., 2014). A major limitation of these species for genetic analysis of reproductive incompatibilities, however, is their age: as *D. melanogaster* diverged from *D. simulans* ∼3 Mya, all of their hybrids are dead or sterile, and genetic analyses involving *D. melanogaster* are limited, under most circumstances, to F_1_ hybrids [but see Sawamura et al. (2000); Masly et al. (2006))]. This problem has been circumvented in part by the discovery of “hybrid rescue mutations”— compatible alleles at otherwise incompatible loci that completely (or nearly so) reverse hybrid lethality (Watanabe, 1979; Hutter and Ashburner, 1987). The existence of these mutations shows that the genetic basis of hybrid lethality is sufficiently simple that hybrid rescue is possible by changing the genotype at any of a small number of loci. In contrast to hybrid lethality, however, F_1_ hybrid males from these crosses are sterile many times over so that no single-locus change in genotype can rescue their fertility (Sawamura, 2000; Sawamura et al., 2000). This implied faster accumulation of hybrid male sterility turns out to be a general feature of *Drosophila* speciation: among hundreds of species pairs, hybrid male sterility evolves earlier than other forms of hybrid incompatibility [*e.g*., hybrid lethality, hybrid female sterility (Wu, 1992; Wu and Davis, 1993, Coyne and Orr, 1997)]. Hybrid male sterility is therefore more likely to contribute to reproductive isolation, better reflects those biological functions that diverge fastest between species and, as discussed below, presents its own set of puzzles. To study hybrid incompatibility genes and phenotypes that evolve early during species divergence clearly requires studying younger species pairs than the *D. melanogaster-D. simulans* hybridization.

Here we review the history, progress, and major results of the genetics of HMS among the younger species of the *D. simulans* clade species— *D. simulans* (Sturtevant, 1919), *Drosophila mauritiana* (Tsacas and David, 1974), and *D. sechellia* (Tsacas and Bächli, 1981). These species diverged from one another ∼250 Kya [(Kliman et al., 2000; McDermott and Kliman, 2008, Garrigan et al., 2012); but see Schrider et al. (2018) for a younger estimated split time, ∼100 Kya], when a presumed *D. simulans*-like ancestor from Madagascar gave rise to *D. sechellia* on the Seychelles archipelago and *D. mauritiana* on Mauritius and Rodrigues islands. While morphologically similar [except for conspicuous differences in male genitalia (Ashburner et al., 2005)], the three *simulans* clade species are now partially reproductively isolated by geography, ecology (Lachaise et al., 1988), sexual isolation (Coyne, 1992a), postmating-prezygotic isolation (Price, 1997), and intrinsic hybrid incompatibility (Lachaise et al., 1986). During the past 40 years, these species have served as important models for the genetic analysis of speciation and species differences, producing many key breakthroughs that have influenced our thinking about speciation genetics. Here we focus on three major and, at the time, surprising observations to emerge from these efforts: the special role of sex chromosomes; the complex genetic architecture; and the interaction of gene flow and selection. Our review comes with two caveats. Rather than attempt a thorough survey of all hypotheses and alternative models, we focus on those that achieved some critical threshold of attention or otherwise guided the direction of research. And rather than present a taxonomically comprehensive review, we focus narrowly on lessons from the *D. simulans* clade species.

## Sex Chromosomes and Hybrid Sterility

The sex chromosomes invariably play the largest role in hybrid sterility or inviability (Coyne and Orr, 1989a). *The rapid evolution of hybrid male sterility (at least in male-heterogametic taxa) is arguably the most significant legacy from the last decade’s probing of Haldane’s rule* (Wu et al., 1996).

Sex chromosomes feature prominently in three strong empirical patterns that characterize speciation. First, Haldane’s rule is the phenotypic observation from species crosses that if one F_1_ hybrid sex is dead, sterile or otherwise unfit, it tends to be the heterogametic (*XY* or *ZW*) sex (Haldane, 1922; Laurie, 1997, Orr, 1997; Schilthuizen et al., 2011, Delph and Demuth, 2016). Haldane’s rule is notable because it holds widely— in insects, birds, fish, mammals, and plants— and appears to be an obligate, intermediate phase in the gradual evolution of complete hybrid incompatibility (Coyne and Orr, 1989b). The fact that Haldane’s rule holds in male- (*XY*) and female-heterogametic (*ZW*) taxa implicates hybrid sex chromosome genotype rather than sex *per se*. Second, the large X-effect is the genetic observation in backcross analyses that the X chromosome has a disproportionately large effect on hybrid incompatibility, given its physical size and gene content (Dobzhansky, 1936; Coyne and Orr, 1989a; Coyne, 1992b; Presgraves, 2008a). To these “two rules of speciation” (Coyne and Orr, 1989a), a third population-genetic observation may be relevant: genetic differentiation between taxa is generally greater on the X chromosome than the autosomes, consistent with reduced interspecific gene flow on the X (Presgraves, 2018). All three patterns have attracted the attention of speciation geneticists as all three involve sex chromosomes, suggesting the possibility of a single, unitary explanation. If we can determine why the X (or Z) chromosome plays a special role in hybrid incompatibility, then we might have a ready explanation for three strong generalizations in speciation. Causes for the special role of sex chromosomes in speciation can be partitioned into (proximate) genetic causes and (ultimate) evolutionary causes which we discuss in turn.

### Genetic Causes

**FIGURE 1:**
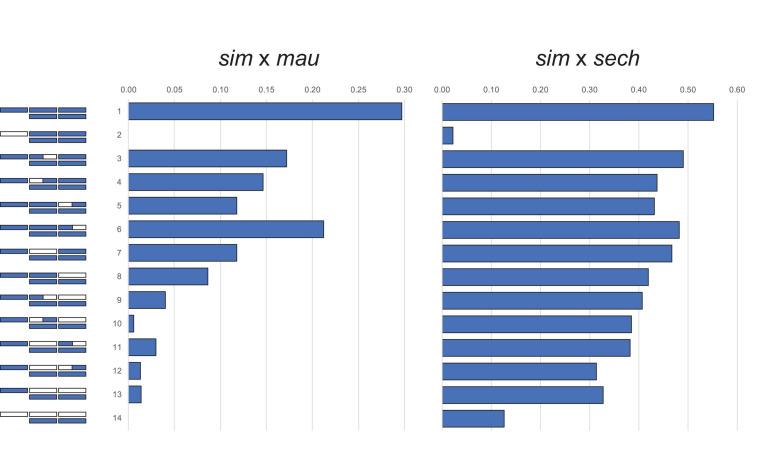
The large X-effect in backcross hybrid male sterility from crosses between *D. simulans* (*sim*) and *D. mauritiana* (*mau*) and between *D. simulans* and *D. sechellia* (*sech*). Bars represent the proportion males with motile sperm. *D. simulans* chromosome segments shown in purple, *D. mauritiana* or *D. sechellia* in white. Figures reproduced from data in Coyne (1984) and Coyne and Kreitman (1986).

Crosses between the three *D. simulans* clade species follow Haldane’s rule: F_1_ hybrid females are fertile but F_1_ hybrid males completely sterile (Lachaise et al., 1986). Backcross analyses of HMS between *D. simulans* (*sim*), *D. mauritiana* (*mau*), and *D. sechellia* (*sech*) show a large X-effect [Figure 1; (Coyne, 1984; Coyne and Kreitman, 1986)]. The X chromosome might have a disproportionately large effect on hybrid fitness problems for any of three genetic reasons: (1) the effect sizes of hybrid incompatibility factors on the X exceeds those of autosomal ones (Coyne and Orr, 1989a); (2) the density of hybrid incompatibility factors on the X exceeds that for the autosomes (Charlesworth et al., 1987; Coyne and Orr, 1989a; Turelli and Orr, 2000; Naveira, 2003); and/or (3) the negative effects of incompatibility alleles are on average recessive in hybrids (Muller, 1940, 1942, Turelli and Orr, 1995, 2000). Under this dominance theory, Haldane’s rule occurs because the *XY* sex, being hemizygous for the X, suffers the full effects of any X-linked hybrid incompatibilities whereas the *XX* sex, being heterozygous, is not. Similarly, the large X-effect occurs because foreign X-linked alleles are always hemizygous in backcross hybrid males whereas autosomal ones are always heterozygous (Wu and Davis, 1993).

**FIGURE 2:**
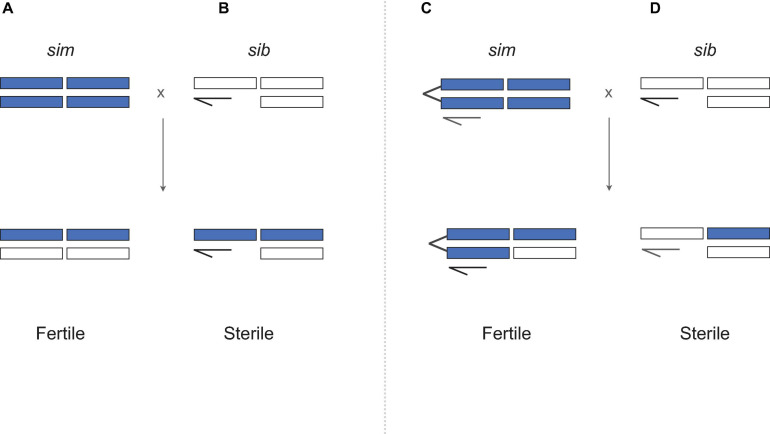
Querying the genetic basis of Haldane’s rule **(A,B)** using the attached-X test. **(C,D)** Attached-X chromosomes correspond to two X chromosomes that have been fused together and now segregate as a single chromosome. When *D. simulans* females carrying an attached-X are crossed to heterospecific males, the female F_1_ progeny inherit the *sim* attached-X and a heterospecific Y chromosome which has no effect in females (sex-determination in *Drosophila* is determined by the number of X chromosomes). Crosses involving attached-X chromosomes show that hybrid males are sterile **(D)** and hybrid females are fertile **(C)** despite having equivalent genotypes **(B** *versus* **C)**. *D. simulans* (*sim*) material is shown in purple, *D. mauritiana* or *D. sechellia* (“*sib*”) material shown in white. Crosses based on Coyne (1985).

In a seminal paper, Coyne (1985) assayed the fertility of “unbalanced” F_1_ hybrid females that are *homozygous* for the *sim* X chromosome (Figure 2). If hybrid sterility in males results from exposure of recessive X-linked incompatibility alleles in hemizygous state, then genotypically equivalent hybrid females should be sterile too. They are not (Figure 2C). One possibility is that X-Y incompatibilities cause sterility in hybrid males but not in attached-X hybrid females, for which the Y is irrelevant for oogenesis. For *sim* and *sech*, however, direct tests for X-Y incompatibilities show that (1) *Y*^*sech*^ does not cause HMS in a *sim* genetic background; and (2) the sterility of *X*^*sech*^/*Y*^*sim*^ males is caused by X-autosome, not X-Y, incompatibilities (Johnson et al., 1992, 1993, Zeng and Singh, 1993). Together, these findings falsify any model in which sex differences in hybrid fitness are solely attributable to sex differences in genotype, as the same genotype sterilizes males but not females— instead hybrid males are sterile because they suffer *different* hybrid incompatibilities than hybrid females. The results lead to two strong conclusions. Hybrid sterility factors are sex-specific, as might be expected given highly sex-specific gametogenic programs (Lindsley and Tokuyasu, 1980); and genetic factors causing hybrid *male* sterility accumulate faster between these species than those causing hybrid *female* sterility (Wu and Davis, 1993; Wu et al., 1996).

Fine-scale genetic analyses, in which small chromosomal segments generally comprising ≤2% of the genome are introgressed between species via recurrent backcrossing, confirm both conclusions: HMS factors accumulate at least ∼10 times faster than hybrid lethal or hybrid female sterility factors (Hollocher and Wu, 1996; True et al., 1996a, Tao et al., 2003a; Masly and Presgraves, 2007, Meiklejohn et al., 2018). These experiments also reveal a new, third conclusion— HMS accumulates between species at least 2.5- to 4-fold faster on the X chromosome than on the autosomes (True et al., 1996a; Tao et al., 2003a, Masly and Presgraves, 2007). This rapid accumulation of X-linked, male-specific hybrid sterility informs long-running debates about the genetic causes of Haldane’s rule and the large X-effect, and refutes the dominance explanation for hybrid male sterility. With these findings in mind, Turelli and Orr (Turelli and Orr, 1995, 2000) formulated a general “composite theory” for Haldane’s rule and the large X-effect. In male heterogametic taxa, Haldane’s rule is expected when

(1) $$R=\tau \,\left(1-g_{x}\right)\,\left(1-g_{x}+g_{x}/d_{0}\right)>1$$

where τ is the ratio of the number of male- to female-specific incompatibilities, *g*_*X*_ is the fraction of all incompatibilities that are X-linked, and *d*_0_ is the mean dominance of incompatibility alleles. (For brevity, we have suppressed some of the formal details of the model; see Turelli and Orr (2000) for the fuller treatment.) Data from the fine-scale genetic analyses suggest, conservatively, that τ ≥ 10 and *g*_*X*_ ≥ 0.4 [(Tao and Hartl, 2003; Masly and Presgraves, 2007); see below]. Estimates of *d*_0_ are not available, but it does not matter: for all *d*_0_ ≤ 1, Haldane’s rule will be observed (*R* ≥ 6). Given the estimates of τ and *g*_*X*_, the large X-effect is similarly inevitable regardless of dominance (Naveira, 2003). Haldane’s rule among the *simulans* clade species can thus be explained entirely by the faster accumulation of factors causing hybrid male *versus* female sterility, whereas the large X-effect can be explained by the particularly fast accumulation of HMS factors on the X chromosome. The question now is why.

### Evolutionary Causes

A simple mutagenic potential model cannot account for the rapid evolution of X-linked HMS: based on gene numbers and locations in *Drosophila*, viability presents a ∼10-fold larger mutational target for interspecific divergence than male fertility (Wu and Davis, 1993; Lindsley et al., 2013), and the X chromosome is neither enriched nor depleted for male fertility-essential or testis-expressed genes (Meiklejohn and Presgraves, 2012; Meisel et al., 2012, Lindsley et al., 2013). We therefore need evolutionary models that can account for the >10-fold excess of factors causing HMS *versus* hybrid lethality (or hybrid female sterility) *and* for the >2.5-fold excess of HMS factors on the X *versus* the autosomes.

#### Faster-Male Evolution

The faster evolution of HMS than hybrid female sterility might occur for one, or a combination, of two reasons. First, genes involved in male reproductive function might evolve faster as a consequence of sexual selection, giving rise to male-specific hybrid incompatibilities (Wu and Davis, 1993; Wu et al., 1996). There is no shortage of evidence for sexual selection on male reproductive signals (Anholt et al., 2020), morphology (*e.g*., genitalia, reproductive tract, and sperm [Miller and Pitnick, 2002; Frazee and Masly, 2015)], fertilization biology (Price, 1997), gene expression (Meiklejohn et al., 2003), and protein sequences (Swanson et al., 2001). Notably, however, the faster-male theory does not necessarily predict faster evolution for the X chromosome. Second, substitutions that affect spermatogenesis and oogenesis could in principle accumulate at similar rates, but spermatogenesis might be more sensitive to developmental disruption than oogenesis because, *e.g*., the elaborate postmeiotic stages of spermatogenesis proceed largely in the absence of transcription (Wu and Davis, 1993), leading to a greater proportion of those substitutions causing male-specific incompatibilities. As often noted (Wu and Davis, 1993; Laurie, 1997, Orr, 1997), a major weakness of both flavors of the faster-male theory— sexual selection and “spermatogenesis is special”— is that they are difficult to reconcile with the ubiquity of Haldane’s rule in female-heterogametic taxa where hybrid females are sterile but males are fertile (Presgraves, 2002; Price and Bouvier, 2002).

#### Faster-X Evolution

The faster-X theory shows that X-linked loci can experience higher rates of adaptive evolution than autosomal loci (Charlesworth et al., 1987). The original model assumes, critically, that adaptation proceeds via the fixation of unique beneficial mutations that increase heterozygous fitness by *sh* and hemi- or homozygous fitness by *s* (where *s* = the selection coefficient and *h* = the dominance coefficient); an equal sex ratio so that the effective population size of the X is 3/4 that of autosomes; and equal germline mutation rates for the two sexes. Then, the ratio of the rate of adaptive substitution on the autosomes and the X is

(2) $$\frac{R_{A}}{R_{X}}=\frac{4\,h}{1+2\,h}$$

and faster-X evolution will occur when unique beneficial mutations are, on average, partially recessive (mean *h* < 1/2). (It is important here to distinguish the dominance of the beneficial effects of adaptive mutations within species *versus* the dominance of any incompatibility effects these mutations might have in species hybrids.) The magnitude of faster-X evolution can be enhanced for mutations that are more beneficial to males than females (Charlesworth et al., 1987). Empirical support the faster-X theory is found in multiple signatures of excess positive selection on the X relative to the autosomes, including more selective sweeps, more genes with histories of recurrent adaptive evolution, and a greater estimated proportion of adaptive substitutions (Meisel and Connallon, 2013).

If HMS arises as an incidental by-product of adaptive evolution within species, then faster-X evolution could help explain the faster evolution of HMS on the X (Charlesworth et al., 1987; Coyne and Orr, 1989a). More precisely, if adaptive substitutions on the X and autosomes have equal probabilities of causing HMS, then the X/A ratio of HMS factors is expected to be equal to the X/A ratio of substitution rates (Naveira, 2003). Instead, the observed X/A ratio for HMS factors between *D. simulans* and *D. mauritiana* (>2.5) *cannot* be explained by the observed X/A ratio for protein-coding sequence divergence [∼1.2; (Begun et al., 2007; Garrigan et al., 2014)]. The X/A ratio of interspecific divergence, however, confounds neutral, slightly deleterious, and adaptive substitutions. Theory shows that faster-X evolution occurs only when adaptation occurs via unique beneficial mutations, not via standing genetic variation or recurrent mutation (Orr and Betancourt, 2001). Therefore, to the extent that faster-X evolution contributes, HMS must be a by-product of the substitution of new rather than recurrent or segregating mutations. Despite these suggestions for a role for faster X evolution, there is an important caveat: while faster-X evolution can result in excess hybrid incompatibilities on the X, it does not on its own cause Haldane’s rule. If, in Eq. 1, τ = 1 and *d*_0_ = 0.5, then hybrid males and hybrid females will be equally fit despite any faster-X evolution (Orr, 1997). In principle, then, faster X evolution could contribute to the large X-effect and/or the high density of HMS factors on the X, but Haldane’s rule will require a separate explanation for the faster accumulation of HMS in general.

#### Misregulation of Gene Expression

Hybrid incompatibilities can result from misregulation of gene expression (Johnson and Porter, 2000; Mack and Nachman, 2016). Disproportionate misexpression of X-linked genes might arise in two ways. First, paralleling protein-coding sequence evolution, the expression levels of X-linked genes evolve faster than autosomal ones (Meisel et al., 2012). We might therefore expect greater misexpression of X-linked genes in hybrids. Second, and more narrowly, heteromorphic sex chromosomes often have chromosome-specific forms of regulation. In *Drosophila*, a dedicated sex chromosome dosage compensation complex (DCC) of proteins and RNAs distinguishes the single male X chromosome from the autosomes and enables hypertranscription of X-linked genes in male somatic cells (Gelbart and Kuroda, 2009). In some lineages, the components of the DCC evolve rapidly (Levine et al., 2007; Rodriguez et al., 2007), raising the possibility that hybrid incompatibilities that disrupt recognition and/or compensation could cause X-linked male-specific lethality [but not, it seems, between *D. melanogaster* and *D. simulans*; (Orr, 1989; Barbash, 2010)]. To explain HMS, we must consider sex chromosome regulation, and its possible disruption, in the male germline. Surprisingly, the regulation of the X in the male germline is still rather poorly understood in *Drosophila*. The facts are that X chromosome expression relative to autosomes is dynamic across stages of spermatogenesis but that, overall, X-linked genes are expressed, on average, at lower levels than autosomal ones. The lower expression of X-linked genes has been attributed to an absence of sex chromosome dosage compensation (Meiklejohn et al., 2011; Meiklejohn and Presgraves, 2012) and/or to a process similar to meiotic sex chromosome inactivation (Hense et al., 2007; Vibranovski et al., 2009, Meiklejohn et al., 2011; Landeen et al., 2016).

There is little indication that *simulans* clade hybrid males experience excess disruption of X chromosome regulation. Genome-wide gene expression analyses in testes from *D. simulans-D. mauritiana* F_1_ hybrids and from introgression hybrids that carry a single X-linked 500-kb region from *D. mauritiana* in a *D. simulans* background show that the proportion of X-linked genes that are misexpressed is roughly half that of autosomal genes that are misexpressed (Moehring et al., 2006b; Lu et al., 2010). If anything, then, the expression of autosomal genes appears more subject to disruption in hybrids. An outsized role of the X in hybrid sterility via gene misregulation therefore requires an excess of *trans*-acting X-linked factors that disrupt the expression of autosomal genes. But, even then, genome-wide studies of gene misexpression in hybrid males (or testes) can be challenging to interpret, for two reasons. First, it is difficult to distinguish gene misregulation that causes sterility *versus* misregulation that results from sterility. Second, and related, it is difficult to distinguish gene misexpression *per se* from the appearance of misexpression caused by the perturbed cell and/or tissue composition of hybrid *versus* parental species testes.

#### Gene Transposition

Dobzhansky (1937) and Muller (1942) both noted that evolutionary changes in the chromosomal locations of genes created the potential for recombinant hybrid genotypes that lack gene copies at both ancestral and transposed positions [see also (Stern, 1936; Werth and Windham, 1991, Lynch and Force, 2000)]. If the transposed gene is essential for male fertility then these double-null recombinant hybrid males would be sterile. The first evidence to support this model comes from certain F_2_-like hybrids between *D. melanogaster* and *D. simulans*: the fertility essential gene, *JYalpha*, is on chromosome *4* in *D. melanogaster* and on chromosome *3* in *D. simulans*; hybrid males homozygous for a *D. simulans* chromosome *4*^*sim*^ in an otherwise *D. melanogaster* background lack *JYalpha* entirely and are thus completely sterile (Muller and Pontecorvo, 1940; Orr, 1992, Masly et al., 2006). With proof of principle established, the question arises as to whether gene transposition might help explain Haldane’s rule and/or the large X-effect. Two genomic patterns characterizing gene transposition in *Drosophila* suggest a potential role (Moyle et al., 2010): there is excess gene transposition “traffic” involving the X chromosome, including both X → autosome and autosome → X gene movements; and transposed genes are disproportionately testis-expressed (Betran et al., 2002; Meisel et al., 2009, Han and Hahn, 2012). The absolute rate of X←→A gene traffic is, however, low relative to the rate of accumulation of HMS: among species lineages in the *D. melanogaster* group, the estimated mean rate of gene movement is ∼2 per million years (Meisel et al., 2009). This estimate suggests ∼0.5 species-specific gene movements in each of the ∼250-Ky old *D. simulans* clade lineages, far too few to account for the many X-linked HMS factors mapped between these species (True et al., 1996a; Tao et al., 2003a, Masly and Presgraves, 2007; Meiklejohn et al., 2018). The contribution of X-linked gene traffic to HMS in the *D. simulans* clade must therefore be negligible.

#### Drive

Meiotic drive refers to the biased transmission of one allele—usually a selfish genetic element— over another from a heterozygous carrier. Drive in the male germline tends to involve two or more loci: a *drive* locus, with wildtype (*D*) and driving (*d*) alleles; a *target*-of-drive locus, with sensitive (*S*) and insensitive alleles (*s*); and a constellation of linked drive-*enhancers* and/or unlinked drive-*suppressors*. In heterozygous *DS*/*ds* males, spermatids bearing sensitive *S* alleles are killed or incapacitated by the action of the driving *d* allele, conferring a transmission advantage to the resistant *ds* haplotype. Whether *d* and *s* invade and spread in a population depends on the frequency of recombination: linkage enables transmission of *d* with *s*, whereas recombination yields “suicide” combinations of *d* with *S*. On autosomes, recombination therefore limits the opportunity for drive. On non-recombining sex chromosomes, however, there is no such limit—*any* factor on the X can drive against *any* target on the Y (and vice versa) without risk of suicide combinations. The resulting sex chromosome drive causes biased progeny sex ratios and reduced fertility, as well as many downstream knock-on consequences (Hamilton, 1967; Jaenike, 2001; Presgraves, 2008b; Meiklejohn and Tao, 2010). Most important among these, for our purposes, are molecular genetic arms races, as sex chromosome drive favors the evolution of resistant alleles at the *target*; *suppressors* at unlinked loci; and counter-resistance and/or suppressor-evasion at *drive* and linked *enhancer* loci. Sex chromosomes are thus subject to recurrent cycles of drive, resistance, suppression, counter-suppression, and so on (Carvalho et al., 1997; Hall, 2004), resulting in the potential accumulation of multiple, divergent, species-specific “cryptic drive” systems— drive loci that persist but in a silenced state.

From this seemingly fanciful premise, Frank (1991) and Hurst and Pomiankowski (1991) suggested that otherwise cryptic drivers might be released or aberrantly expressed in the naïve genetic backgrounds of species hybrids, with mutual destruction of X- and Y-bearing spermatids causing sterility that maps disproportionately to sex chromosomes (Frank, 1991; Hurst and Pomiankowski, 1991). Theirs was a radical suggestion, as most speciation geneticists at the time preferred to invoke classical neo-Darwinian phenomena, like genetic drift and ecological adaptation, and eschewed the seemingly exotic ones, like meiotic drive. Moreover, if meiotic drive causes Haldane’s rule and the large X-effect— the “two rules of speciation”— then drive must be far more ubiquitous than previously supposed. And, not least, some speciation geneticists had already looked for evidence of cryptic drive unleashed in hybrids but found none (Coyne, 1986). For these reasons, skeptics battered the drive theory for its implausibility and lack of evidence (Coyne et al., 1991; Johnson and Wu, 1992, Charlesworth et al., 1993; Coyne and Orr, 1993).

But the drive theory is enjoying a resurgence due to recent findings in mammals (Davis et al., 2015; Kruger et al., 2019, Rathje et al., 2019) and *Drosophila* (Orr and Irving, 2005; Phadnis and Orr, 2008, Zhang et al., 2015), including the *D. simulans* clade species. First, *D. simulans* harbors two well-characterized cryptic sex chromosome drive systems. The Paris drive system involves two co-drivers on the X chromosome, one a segmental duplication containing six genes and the other an allele of the *HP1D2* gene, a rapidly evolving member of the *HP1* heterochromatin protein family (Helleu et al., 2016). The Paris drivers are usually suppressed by resistant Y chromosomes and by multiple loci scattered across the autosomes (Courret et al., 2019; Helleu et al., 2019). The Winters drive system involves a new, lineage-restricted chimeric gene, *Distorter on the X* (*Dox*), that is usually suppressed by an autosomal retroduplicate, *Not-much-yang* (*Nmy*), which silences *Dox* expression by producing *Dox*-matching endogenous small interfering RNAs (Tao et al., 2007a,b, Lin et al., 2018). In a recent genetic analysis of X-linked HMS, we uncovered additional evidence for a novel cryptic X chromosome drive system in *D. mauritiana* (Meiklejohn et al., 2018). These discoveries confirm a key requirement of the drive theory, namely that closely related species accumulate divergent multilocus systems of cryptic drive.

A second requirement of the theory is that drive contributes to the evolution of HMS. Two observations are suggestive here. One is that genetic introgression of the *Too much yin* (*Tmy*) region on *3R* of *D. mauritiana* into *D. simulans* releases cryptic drive and, along with other linked factors, contributes to HMS (Tao et al., 2001). Another is that the *D. mauritiana* allele of the X-linked gene, *OdysseusH* (*OdsH*) (Ting et al., 1998), contributes to HMS in a *D. simulans* genetic background, and the OdsH^*mau*^ protein binds repetitive DNA sequences on the *D. simulans Y*^*sim*^ chromosome but *not* its own *D. mauritiana Y*^*mau*^ chromosome (Bayes and Malik, 2009). While there is no evidence that *OdsH* currently causes drive, it is easy to imagine a history in which *Y*^*mau*^ evolved resistance to *Ods*^*mau*^-mediated drive by shedding target sequences, while the naïve *Y*^*sim*^ retained them. Similar and more direct evidence links drive to HMS in two other *Drosophila* hybridizations (Orr and Irving, 2005; Phadnis and Orr, 2008, Zhang et al., 2015). These suggestive observations from the *simulans* clade species, and the more direct evidence from other species pairs, support the second key requirement that drive can contribute to HMS. The question now is not whether meiotic drive contributes to HMS but to what extent.

#### Satellite DNAs

Haldane’s rule and the large X-effect have never been short of competing explanatory hypotheses. We nevertheless hazard another, admittedly speculative, hypothesis here: the rapid evolution of HMS in the *D. simulans* clade could involve the rapid divergence of repetitive satellite DNA sequences (satDNAs), their regulation, and/or their functional effects. The notion that satDNAs contribute to hybrid incompatibility and speciation is not new (Rose and Doolittle, 1983; Ferree and Prasad, 2012, Sawamura, 2012; Gallach, 2014). But three recent findings from the *D. simulans* clade species and their close relative, *D. melanogaster*, are consistent with a role for satDNAs in the rapid evolution of HMS on sex chromosomes.

First, the sequences, copy numbers, genomic compositions, and chromosomal distributions of simple and complex satDNAs are strikingly different between the *D. simulans* clade species (Jagannathan et al., 2017; Sproul et al., 2020). Cytological analyses reveal that large blocks of [AACAAAC]_*n*_ are detectable in *D. mauritiana* on chromosomes 2 and 3, in *D. simulans* on the Y chromosome, and in *D. sechellia* not at all (Jagannathan et al., 2017). Two complex satDNA families, in particular, show considerable turnover between the *D. simulans* clade species: the *1.688*g/cm^3^ (Hsieh and Brutlag, 1979) and *Rsp-like* (Larracuente, 2014) satellites. In heterochromatic pericentromeric regions, large (Mb-scale) blocks of satDNAs reside in species-specific locations: *1.688* blocks are X-linked in *D. sechellia* but autosomal in *D. simulans* and *D. mauritiana*, whereas *Rsp-like* blocks are X-linked in *D. simulans*, autosomal in *D. sechellia*, and absent altogether from *D. mauritiana* centromeres (Sproul et al., 2020). High quality genome assemblies provide additional comprehensive, fine-scaled evidence for species-specific distributions of blocks of satDNA arrays in heterochromatic regions and small islands of satDNAs in euchromatic regions (Sproul et al., 2020; Chakraborty et al., 2021).

Second, euchromatic islands of satDNAs, including both *1.688* and *Rsp-like*, are significantly enriched on the X chromosome (Hsieh and Brutlag, 1979; Kuhn et al., 2012, Garrigan et al., 2014; Sproul et al., 2020). In the euchromatin, small (≤kb-scale) satDNA islands are enriched on the X relative to autosomes 15-fold in *D. simulans*, 29-fold in *D. mauritiana*, and 51-fold in *D. sechellia* (Chakraborty et al., 2021). This X chromosome enrichment results from expansion of satDNA islands on the X rather than loss from the autosomes. While some, presumably older, satDNA islands, like *1.688*, are shared as homologous array loci among species, newer satDNA islands, like *Rsp-like*, tend to be species-specific (Sproul et al., 2020).

Third, and critically, satDNA-derived small RNAs are essential for male fertility in *D. melanogaster* (Mills et al., 2019). In particular, depletion of small RNAs from the highly abundant AAGAG tandem repeat results in defective histone-to-protamine transition during spermiogenesis (Mills et al., 2019). The exchange of canonical histones for sperm-specific protamines facilitates remodeling of sperm pronuclei into compact, non-nucleosomal structures that are ∼200-fold smaller in volume (Rathke et al., 2007). Processing of satDNAs during this radical chromatin remodeling appears to be susceptible to disruption. In *D. melanogaster*, for example, the autosomal meiotic drive gene complex *Segregation Distorter* (*SD*) achieves a >95% transmission advantage from heterozygous *SD/* + males by disrupting the histone-to-protamine transition for + -bearing spermatids that have large blocks of *Rsp* satDNA (Wu et al., 1988; Gingell and McLean, 2020). In sterile F_1_ hybrid males between *D. simulans* and *D. mauritiana*, sperm nuclei show an age-dependent de-condensation phenotype (Kanippayoor et al., 2020), suggestive of compromised chromatin integrity, but whether specific misregulation of satDNAs is involved is unknown. [Spermatogenesis in sterile F_1_ hybrid males from the reciprocal cross, in contrast, arrests during premeiotic stages (Kulathinal and Singh, 1998)].

Overall, then, the satDNA hypothesis merits our attention because satDNA composition evolves quickly, satDNAs are enriched on the X, and disruption of satDNA regulation can disrupt male fertility. One obvious way to distinguish among these several hypotheses— faster male, faster X, drive, satDNAs, *etc*.— is to determine the molecular genetic basis of HMS. Our prospects for characterizing the molecular genetic basis of HMS depends on its genetic architecture, to which we turn next.

## Genetic Architecture of Hybrid Male Sterility

*When an introgressed segment that produces sterility is partitioned by recombination into shorter segments, sterility vanishes* (Naveira and Maside, 1998). *(H)ybrid sterility between incipient species is largely due to strong epistasis between genes of minor or no effect individually* (Cabot et al., 1994).

The Dobzhansky-Muller (DM) model provides a simple, intuitively satisfying solution to the puzzle of how hybrid incompatibility might evolve, and it is supported by an abundance of genetic data from a variety of systems, including yeast, worms, flies, butterflies, fish, mouse, *Arabidopsis*, *Mimulus*, and more (Johnson, 2010; Presgraves, 2010). But the power of the DM model has not translated into routine identification of hybrid incompatibility genes. Aside from two notable successes (see below), the search for hybrid incompatibility genes has been hampered in several ways. First, reproductive isolation gets in the way, often prohibiting the creation and/or maintenance of desired genotypes. Second, hybrid incompatibilities are often polymorphic: the effect sizes, or even the existence, of genetic incompatibility alleles mapped between two particular strains may not hold for others. Third, despite the appeal of the simple two-locus DM model, hybrid incompatibilities almost always involve more than two factors— hybrids are sterile because they have the “wrong” genotype at ≥ 3 loci. Compounding these challenges, the “genetic architecture” of HMS loci appears to differ from that of other kinds of hybrid incompatibility.

### Genetic Architecture

**FIGURE 3:**
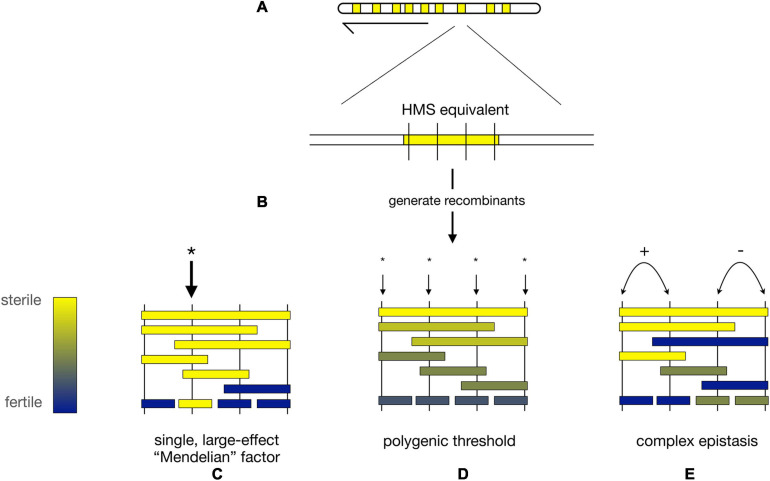
Alternative models for the genetic architecture of HMS. **(A)** Hypothetical map of nine X-linked regions from *D. mauritiana* (*mau*) that each cause strong HMS when introgressed into a *D. simulans* (*sim*) genetic background (“HMS equivalents,” yellow). **(B)** Fine-scale recombination mapping is used to dissect the genetic basis of each HMS equivalent region. **(C)** HMS regions may contain a single *mau* factor of large phenotypic effect (* = location of HMS factor). **(D)** Under the polygenic threshold model, multiple, interchangeable *mau* factors individually contribute to HMS; complete HMS occurs when a sufficient, threshold number of polygenic HMS factors is present simultaneously. **(E**) Under the complex epistasis model, two (or more) loci interact to determine the HMS phenotype. In the figure, there are two pairs of adjacent, epistatically interacting loci. The left pair of loci shows synergistic epistasis (+) in which two *mau* alleles interact to produce stronger HMS than the sum of their individual effects. The right pair of loci shows antagonistic epistasis (−) in which two *mau* alleles interact to produce weaker HMS than the sum of their individual effects.

At the level of whole chromosomes, there are many HMS loci, few hybrid lethals, and even fewer hybrid female steriles (Hollocher and Wu, 1996; True et al., 1996a, Tao and Hartl, 2003; Masly and Presgraves, 2007, Meiklejohn et al., 2018). At the level of individual loci, the distribution of effect sizes for HMS and hybrid lethality differs as well. (We omit hybrid female sterility from this discussion because so little information is available). Numerous hybrid lethality factors of large effect have been identified between *D. melanogaster* and *D. simulans* (Sawamura and Yamamoto, 1997; Barbash et al., 2003, Presgraves et al., 2003; Tang and Presgraves, 2009, Phadnis et al., 2015) and several between *D. mauritiana* and its sister species (True et al., 1996a; Masly and Presgraves, 2007, Cattani and Presgraves, 2009). In all of these cases, hybrid lethality could be localized to an individual gene or repetitive DNA element. These findings, plus early genetic mapping results from the *D. simulans* clade species (Coyne and Charlesworth, 1986, 1989, Perez et al., 1993), nurtured expectations that HMS loci might also have large effects (Figures 3A–C).

There were, however, early indications to the contrary. Genetic analyses between *Drosophila buzzatti* and *Drosophila koepferae* showed that interspecific introgressions on the autosomes were generally male-fertile when <30% the length of the chromosome but invariably male-sterile when >35% (Naveira and Fontdevila, 1985, 1986, 1991). No single locus caused HMS unless co-introgressed with some minimum number of additional HMS loci. The genetic basis for HMS appears similarly diffuse between *D. mauritiana* and *D. simulans* (Naveira, 1992). On the X chromosome, for instance, a ∼500-kb region from *D. mauritiana* containing *OdsH* causes strong HMS in a *D. simulans* genetic background only when co-introgressed with other (unknown) factors (Perez and Wu, 1995). On the autosomes, *D. mauritiana* introgressions into *D. simulans* uncovered ∼19 HMS loci on chromosome 3: all but one have modest effects on male fertility, and complete HMS requires the combined effects of multiple loci (Tao et al., 2001, 2003b).

Two models have been proposed to describe these observations. First, the *polygenic threshold* model posits that HMS results when a sufficient number of interchangeable, small-effect factors are co-introgressed together [Figure 3D; (Naveira and Maside, 1998)]. Under this model, the identity of the particular HMS loci involved matters less than the cumulative effects of the multiple, independent HMS factors. Second, the *complex epistasis* model posits that HMS is caused by synergistic epistatic interactions among co-introgressed conspecific alleles that are together incompatible with heterospecific factors (Figure 3E). Under this model, the genotypes at particular loci matters, as two (or more) weak alleles interact to produce an HMS effect greater than the sum of their individual effects (Cabot et al., 1994; Palopoli and Wu, 1994, Wu and Palopoli, 1994; Perez and Wu, 1995, Davis and Wu, 1996; Wu and Hollocher, 1998).

**FIGURE 4:**
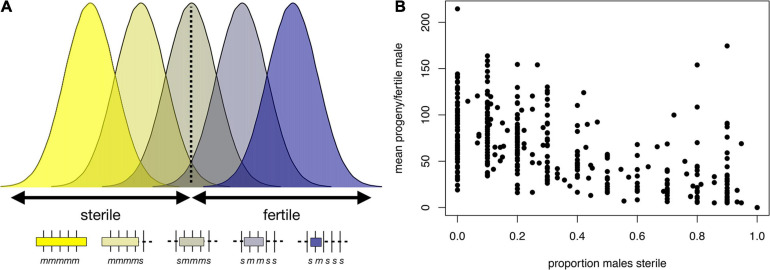
Polygenic threshold model of HMS (adapted from Lienard et al., 2016). **(A)** The plot shows the distribution of a hypothetical quantitative trait, fertility potential, for five hypothetical introgression genotypes shown beneath the *x*-axis. For each genotype, the length of a *D. mauritiana* introgression is represented by the length of the open bar; the genotypes at five markers (vertical tick marks) are indicated by *m* and *s*, for *D. mauritiana* and *D. simulans*, respectively; and the average fertility of males is indicated by the color of the bar. The largest introgression (*mmmmm*) is completely male-sterile, whereas the smallest introgression (*smsss*) is completely fertile. For intermediate introgression genotypes, some proportion of males produce no progeny (those falling below the threshold) whereas others produce >0 progeny. This polygenic threshold model suggests a correlation between the proportion of sterile males associated with a particular introgression genotype and the mean number of progeny produced by their fertile brothers. **(B)** Experimental data on *D. mauritiana* X-chromosome introgressions in a *D. simulans* genetic background show the predicted correlation (Spearman’s ρ = −0.91, *P* < 0.0001) between the proportion of sterile males and the mean number of progeny among fertile males (for data and details see Meiklejohn et al., 2018).

**FIGURE 5:**
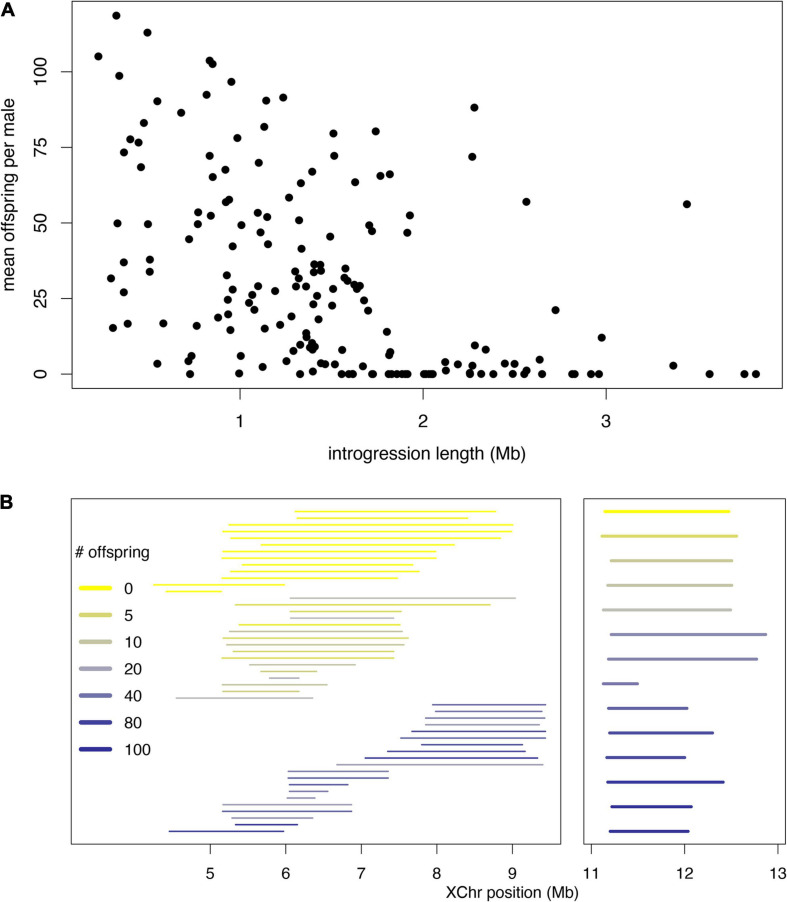
Data from fine-scale genomic analysis of HMS. X chromosome segments from *D. mauritiana* were introgressed via >25 generations of backcrossing into a *D. simulans* genetic background and assayed for fertility by crossing individual introgression males with three virgin *D. simulans* females for seven days (for data and details see Meiklejohn et al., 2018). For each introgression, ≥ 10 clonal males were phenotyped. **(A)** In genomic coordinates *X*:4–13 Mb, the fertility of all introgressions shows a strong negative correlation with length, with very few fertile introgressions >2 Mb. **(B)** Introgressions consistent with the polygenic threshold model. No single *D. mauritiana* locus between coordinates *X*:5–9 Mb is sufficient to cause HMS, but longer introgressions can. The median length of sterile introgressions (top group, yellow) is 1.7-fold greater than that for fertile introgression (bottom group, blue); Wilcoxon test *P* ∼ 0.0001. Between both *X*:4–6 Mb and *X*:11–12 Mb (right panel), small sterile introgressions can, however, fall within overlapping, larger fertile introgressions, consistent with complex epistasis.

These models are not mutually exclusive, and indeed there is evidence consistent with both. We highlight examples gleaned from our recent X chromosome-wide introgression analysis of HMS. To map HMS factors, we assayed *D. mauritiana* introgressions in a *D. simulans* genetic background, delimited introgression size precisely using genotyping-by-sequencing, and measured fertility in replicate males for each genotype (Meiklejohn et al., 2018). Two features of the data are consistent with the polygenic threshold model. First, the polygenic threshold model predicts a correlation between penetrance (the proportion of males with a particular genotype that show the HMS phenotype) and the mean number of progeny for isogenic brothers that sired any progeny (Figure 4A). This prediction is supported by the introgression data (Figure 4B; Spearman’s ρ = −0.56, *P* < 0.0001). Second, within a 9 Mb interval in the middle of the X chromosome, male fertility appears to be a declining function of the amount of introgressed *D. mauritiana* sequence that is largely independent of chromosomal location, with a rough threshold length of ∼2 Mb, beyond which most introgressions are male-sterile (Figure 5A; Spearman’s ρ = −0.56, *P* < 0.0001). Of course, longer introgressions might cause HMS because they are more likely to capture a large-effect HMS factor. But with our high-coverage introgression map, we should observe at least some small introgressions that also capture large-effect HMS factors. We do not. Thus, for the medial ∼50% of the X chromosome, there appears to be no major effect factor that causes HMS on its own. These observations suggest that there must be functional divergence at a very large number of sites that each contribute, if weakly, to HMS.

At a smaller (sub-Mbp) scale, however, other features of the data implicate complex epistasis. In particular, sterile introgressions can be spanned by tiling paths of fertile introgressions or even completely overlapped by larger, fertile introgressions (Figure 5B). Similar observations obtain for autosomal introgressions between *D. mauritiana* and *D. simulans* (Tao et al., 2003b). These findings are difficult to reconcile with the polygenic threshold model: for any sterile introgression, a longer completely overlapping introgression should also be sterile. One explanation is that HMS alleles experience antagonistic interactions in which a small introgression causes HMS whereas co-introgression of additional factors suppresses HMS. Thus, evidence exists for both synergistic and antagonistic forms of complex epistasis (Figure 3E; Wu and Palopoli, 1994; Wu and Hollocher, 1998, Tao et al., 2003b). A polygenic architecture with two flavors of complex epistasis has two implications. The biological implication is that, in hybrids, HMS alleles do not behave as loss-of-function mutations at male fertility-essential genes. The practical implication is that individual HMS loci will be refractory to molecular identification.

### HMS Genes

Despite the practical challenges, the molecular identities of HMS factors have been established in two cases in the *D. simulans* clade species. The first is the well-known, well-characterized X-linked HMS gene, *OdsH* (Ting et al., 1998). By itself, the *D. mauritiana* allele of *OdsH* causes sperm motility defects in ∼50% of introgression male carriers (Perez and Wu, 1995). Complete HMS (no sperm motility) occurs only when other factors are co-introgressed (Perez and Wu, 1995). Nevertheless, X chromosome-wide genetic analyses suggest that *OdsH* may be the HMS factor of the single largest individual effect (Meiklejohn et al., 2018). *OdsH* encodes a testes-expressed protein with a highly diverged DNA-binding homeodomain (Ting et al., 1998). While rapid evolution at *OdsH* was first hypothesized to result from sexual selection, its localization to Y chromosome satDNAs implicates genetic conflict [see above; (Bayes and Malik, 2009)].

The second identification of HMS factors comes from a 9-kb interval on chromosome arm *3R* that contains just four protein-coding genes (Araripe et al., 2010). This *HMS1* region has a very large effect (∼200 progeny for the *D. simulans* allele *versus* ∼2 for the *D. mauritiana* allele), making it a promising candidate for molecular characterization (Araripe et al., 2010). Transgenic experiments reveal, however, that even within this 9-kb region the genetic architecture of HMS is complex (Lienard et al., 2016). Transgenes carrying two different genes— *agt* and *Taf1*— each recover substantial (if not full) male fertility, implicating both in HMS. Both genes encode unrelated DNA binding and/or modifying proteins, but neither has signatures of recurrent positive selection. Chimeric transgenes that combine regulatory sequences from one species and coding sequences from the other at both genes similarly rescued fertility, further suggesting that multiple *D. mauritiana* substitutions distributed across coding and non-coding regions of both genes may be required for HMS.

Work on the genetic architecture of HMS— from large-scale high-resolution genetic analyses to the molecular identification of genes— supports a polygenic basis with additional evidence of complex (synergistic *and* antagonistic) epistasis. These inferences are necessarily based on analyses that seek to isolate individual HMS factor(s) in introgression hybrid male genotypes. The genetic basis of HMS in introgression hybrid males may of course differ from that in F_1_ hybrid males, as they have different genotypes. But if incompatibilities that conform to the polygenic threshold model are abundant, we may safely posit that F_1_ hybrid males are sterile due to the combined effects of very many, individually weak, HMS factors. If gene flow nevertheless occurs via fertile hybrid females, however, then many individually weak HMS factors will be exposed to selection in backcross or advanced backcross hybrid males. It is important to appreciate that many factors deemed to have “weak” phenotypic effects in the laboratory are readily detectable by natural selection.

## Complex Speciation With Gene Flow

*Species in sexual cross-fertilizing organisms are defined as groups of populations which are reproductively isolated to the extent that the exchange of genes between them is absent or so slow that the genetic differences are not diminished or swamped* (Dobzhansky, 1944). *(D)iverging genomes during (or even after) speciation can be quite “porous” with respect to gene flow at non-speciation loci* (Wu, 2001).

Under simple allopatric speciation, populations isolated by geography eventually and incidentally evolve intrinsic reproductive incompatibility, a scenario that “appears so plausible that it hardly seems worth documenting” (Coyne and Orr, 2004). The three species of the *D. simulans* subcomplex would seem to be strong and obvious candidates for allopatric speciation via dispersal: they are believed to have originated on different Indian Ocean islands (Madagascar, the Seychelles, and Mauritius); *D. simulans* has never been collected on Mauritius (David et al., 1989); and, until recently, *D. simulans* had not been collected on the same islands of the Seychelles as *D. sechellia* (Lachaise et al., 1988). In geographic isolation, the three species have evolved ecological, sexual, postmating-prezygotic, and postzygotic barriers (Lachaise et al., 1986, 1988; R’Kha et al., 1991; Coyne, 1992a; Coyne and Charlesworth, 1997; Price, 1997). Early multi-locus population genetic analyses among the three species were, as expected, consistent with a simple model of isolation without gene flow (Kliman et al., 2000; Nunes et al., 2010).

**FIGURE 6:**
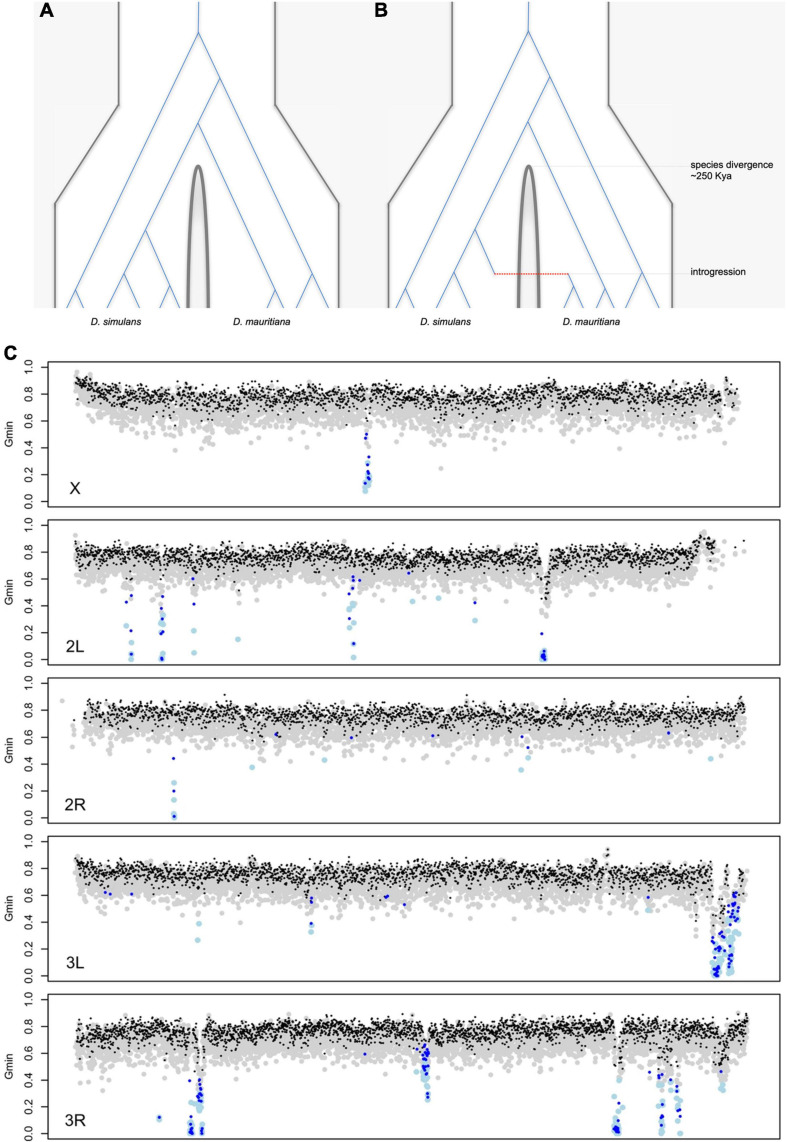
Hypothetical genealogical histories of multiple sequences sampled from two species under **(A)** simple allopatric speciation with no gene flow and **(B)** complex speciation with gene flow. Under simple allopatric speciation, all coalescent events among any two gene copies from the different species must predate the species divergence time. Under complex speciation, coalescent events between any two gene copies from the different species can postdate the species divergence time, as represented by the single introgression event (red). **(C)** A genome-wide scan for introgression in population samples from *D. simulans* (*n* = 20) and *D. mauritiana* (*n* = 10). The *G*_*min*_ statistic was used to identify haplotypes with interspecific distances too low to be consistent with a simple allopatric speciation history (Geneva et al., 2016). Each gray (black) dot corresponds to a 5-kb (10-kb) genomic window consistent with a simple allopatric history, and each light blue (dark blue) dot corresponds to a 5-kb (10-kb) genomic window for which the simple null model is statistically rejected. Introgression is significantly underrepresented on the X chromosome (Meiklejohn et al., 2018).

There are now good reasons to doubt simple allopatric histories for these species. For *D. sechellia* and *D. simulans*, the evidence is direct: the two species now co-occur on a subset of the Seychelles (likely via human introductions), and hybrid males have been collected in the field (Matute and Ayroles, 2014; Navascues et al., 2014). For *D. mauritiana* and *D. simulans*, the first hints of gene flow came from mitochondria: ∼88% of *D. mauritiana* flies carry a *D. simulans*-like mitochondrial haplotype estimated to have introgressed ∼4,500 years ago (Solignac and Monnerot, 1986; Solignac et al., 1986, Satta and Takahata, 1990, Ballard, 2000a,b, Nunes et al., 2010). Genomic data have confirmed nuclear gene flow among all three species pairs. Simple (allopatric) speciation without gene flow predicts that the genealogical histories of all loci should be compatible with a single species divergence time (Figure 6A). The genomes of the three *D*. *simulans* clade species, however, present clear evidence for complex speciation with gene flow resulting in discrepant, reticulated genealogical histories (Figure 6B). Three different analyses, leveraging different (albeit overlapping) features of the data, estimate similar amounts of introgressed foreign material (2–5%) among the three species (Garrigan et al., 2012; Meiklejohn et al., 2018, Schrider et al., 2018). These findings underscore the limits of population genetic surveys at a small number of loci to detect introgression and contribute to the increasing evidence that gene flow is a common feature of divergence between closely related species, even for species pairs that are geographically allopatric (Mallet, 2005; Seehausen et al., 2014; Mallet et al., 2016).

Several features of the natural introgressions are informative. First, the introgressed haplotypes stand out from the genomic background for having aberrantly low interspecific sequence distances (Figure 6B). Second, the introgressed haplotypes show evidence of gradual erosion by recombination. The estimated lengths of introgressions depend both on local chromosomal recombination rate (*e.g*., longer introgressions tend to reside in low-recombination regions) and time-in-residence [*e.g*., older introgressions tend to be shorter; (Meiklejohn et al., 2018)]. Third, foreign introgressed material is two- to four-fold under-represented on the X chromosome [Figure 6C; (Garrigan et al., 2012; Meiklejohn et al., 2018)]. Between *D. mauritiana* and *D. simulans*, there is only one (∼130 kb-long) recent introgression on the X *versus* 47 on the autosomes [Figure 6C; (Meiklejohn et al., 2018)]. This X *versus* autosome difference in introgression density cannot be explained by, *e.g*., male-mediated admixture (F_1_ hybrid males are sterile so that all gene flow must be via fertile F_1_ hybrid females), nor by chromosomal differences in recombination rate (True et al., 1996b). The simplest interpretation is that X-linked material is less exchangeable between species. To introgress, compatible foreign alleles must first survive selection against genetically linked alleles that are incompatible (or otherwise locally maladaptive) and then escape from their deleterious chromosomal backgrounds by recombination (Bengtsson, 1985). Both are more difficult on the X chromosome, as the greater efficacy of selection on the X eliminates foreign material more quickly than on autosomes, *and* the higher density of hybrid incompatibilities on the X limits the opportunity to escape via recombination (Muirhead and Presgraves, 2016; Fraisse and Sachdeva, 2020).

The existence of interspecific introgression raises the question of what kinds of alleles *do* escape to persist in a foreign genetic background. Are most interspecific introgressions neutral (functionally equivalent) alleles? Or are interspecific introgressions enriched for globally adaptive alleles? At least three introgressions show signatures of positive selection. First, a ∼200-kb region on chromosome arm *3R* has introgressed from *D. simulans* into *D. sechellia*, experienced a partial sweep in *D. simulans* and a complete sweep in *D. sechellia* (Garrigan et al., 2012; Brand et al., 2013, Schrider et al., 2018). The precise target of selection remains unclear. Second, the ∼130-kb haplotype on the X chromosome that has introgressed between *D. simulans* and *D. mauritiana* shows a large, partial sweep in *D. simulans* and a massive, ∼550-kb complete sweep in *D. mauritiana* (Nolte et al., 2013; Garrigan et al., 2014, Meiklejohn et al., 2018). Most intriguing, this introgressed interval spans the cryptic meiotic drive genes, *Dox*, and its parent gene, *Mother of Dox* (*MDox*) (Tao et al., 2007a,b). We hypothesize that *Dox* (and/or *MDox*) swept to high frequency in its native background before being suppressed, then migrated between species where, released from suppression in the new genetic background, it swept to high frequency again, resulting in parallel selective sweeps (Meiklejohn et al., 2018). Last, the introgression of a *D. simulans* mitochondrial haplotype into *D. mauritiana* appears to be non-neutral (Aubert and Solignac, 1990; Meany et al., 2019). These findings suggest that the most conspicuous signals of introgression correspond to loci favored globally by selection. However, the relative contributions of selection- *versus* drift-mediated introgression remains to be determined.

Overall, our findings imply that the interplay of gene flow and selection has shaped the genomic distribution of introgression. We do not know which form of reproductive isolation— geographic, ecological, sexual, postmating-prezygotic, or hybrid incompatibility— was most important during the history of speciation and admixture among these species. But there are compelling reasons to believe that HMS is among the important barriers to gene flow. For one, there is more HMS on the X chromosome and, consequently, less introgression on the X (Tao et al., 2003a; Masly and Presgraves, 2007, Garrigan et al., 2012; Meiklejohn et al., 2018). For another, the one region of the X chromosome where introgression has occurred is, conspicuously, where HMS is weak or absent (Meiklejohn et al., 2018). These two findings suggest that HMS has impeded X-linked introgression *except* for the one chromosomal region lacking HMS. Of course, not knowing the historical order of events, it is possible that the reverse is true: selection-driven introgressions may have shaped the genomic distribution of HMS. For instance, drive-mediated introgression of the *MDox-Dox* haplotype between species may have reduced local interspecific divergence and, incidentally, dampened the local accumulation of HMS (Meiklejohn et al., 2018). If true, it would imply that a drive-mediated trans-species sweep attenuated the evolution of HMS. This scenario highlights an implicit assumption of the drive theory, namely, that drive can contribute to divergence and HMS between strictly allopatric species. For species connected by gene flow, however, drive can introgress between species and erase local divergence. Furthermore, the introgression of a drive element creates additional pressure for any suppressors to follow (Crespi and Nosil, 2012). The role of drive in HMS is therefore contingent on the interplay of drive and gene flow.

## Conclusion

The *D. simulans* clade species have been at the forefront of modern speciation genetics for over 40 years. While many puzzles remain unsolved, many of the successes have offered important lessons. From genetic analyses, we have learned that HMS in the *D. simulans* clade accumulates faster on the X chromosome. Why this is the case is still unresolved. The drive theory has been revived as a potential explanation, fueled by the discovery of multiple cryptic drive systems and by direct evidence for a role for drive in hybrid sterility (Tao et al., 2001; Orr and Irving, 2005, Phadnis and Orr, 2008; Zhang et al., 2015). But if HMS is primarily the result of drive, then the many HMS factors separating these species implies an extraordinary frequency of drive in the history of these species and/or an extraordinary proliferation of enhancer and suppressor loci associated with a smaller number of drive systems. We should also be clear that, with few exceptions (Moehring et al., 2006a; Good et al., 2008, Phadnis, 2011; Bi et al., 2015), genetic analyses have not yet established whether the X (or Z) has a relatively higher density of hybrid incompatibilities in other taxa. And, of course, Haldane’s rule and the large X effect may have different causes in different taxa. A composite model may well prevail (Wu and Davis, 1993), albeit one with different emphases than originally imagined. Explanations based on sexual selection, for instance, appear to have ceded ground to those based on genetic conflict.

From fine-scale genetic analyses, we have learned that the genetic architecture of HMS is best described by polygenic threshold and complex epistasis models with very few large-effect HMS factors separating the species. For both the X and the autosomes, apparently large-effect loci correspond to “HMS equivalents” (Tao et al., 2003b) that can be genetically decomposed into multiple factors with incomplete penetrance and/or sub-detectable phenotypic effects. This genetic architecture has important practical implications. First, the preponderance of individually weak-effect HMS factors hinders their genetic isolation and experimental validation (Wu and Palopoli, 1994). The HMS “success stories” (*OdsH*, *JYalpha*, and *Overdrive*) are not a random sample of HMS genes— they are large-effect outliers. In this sense, the genetics of speciation and the genetics of adaptation have, for similar practical reasons, both accumulated well-known, possibly unrepresentative, success stories involving large-effect, Mendelian factors (Rockman, 2012). Second, a polygenic architecture implies that the ∼15 HMS equivalents between *D. simulans* and *D. mauritiana* are underpinned by hundreds of substitutions with modest negative effects on male fertility. It is important to remember, however, that even “weak-effect” HMS factors, as determined by lab-based genetic analyses, are nonetheless readily detectable by natural selection in admixed populations and thus determine the level and genomic distribution of interspecific gene flow.

From population genomics analyses, we have learned that *geographically* allopatric species are not necessarily *genetically* allopatric. It appears that the inter-island dispersals of *D. simulans*-like ancestors to Mauritius and to the Seychelles ∼250,000 years ago were not unique events, as evidenced by recent nuclear and mitochondrial introgression. The resulting genomic distribution of introgression, however, is clearly shaped by the interplay of negative selection against incompatible and locally maladaptive alleles and positive selection for globally adaptive ones. Our findings reveal that selection against HMS disproportionately limits introgression on the X, whereas adaptation (Brand et al., 2013; Schrider et al., 2018) and drive (Meiklejohn et al., 2018) have enabled introgression. Now that we know that admixture has occurred, we can leverage the functional genetic and population genomics resources of the *D. simulans* clade species to further deconstruct the interaction of gene flow and natural selection during speciation.

## Author Contributions

DP and CM contributed equally to the preparation of the manuscript. Both authors contributed to the article and approved the submitted version.

## Conflict of Interest

The authors declare that the research was conducted in the absence of any commercial or financial relationships that could be construed as a potential conflict of interest.

## References

[B1] Anholt R. R. H. O’Grady P. Wolfner M. F. Harbison S. T. (2020). Evolution of reproductive behavior. *Genetics* 214 49–73. 31907301 10.1534/genetics.119.302263 PMC6944409

[B2] Araripe L. O. Montenegro H. Lemos B. Hartl D. L. (2010). Fine-scale genetic mapping of a hybrid sterility factor between *Drosophila* simulans and *D. mauritiana*: the varied and elusive functions of speciation genes. *BMC Evol. Biol.* 10:385. 10.1186/1471-2148-10-385 21144061 PMC3020225

[B3] Ashburner M. Golic K. G. Hawley R. S. (2005). *Drosophila: A Laboratory Handbook. Cold Spring Harbor.* New York: Cold Spring Harbor Laboratory Press.

[B4] Aubert J. Solignac M. (1990). Experimental evidence for mitochonrial DNA introgression between *Drosophila* species. *Evolution* 44 1272–1282. 10.1111/j.1558-5646.1990.tb05231.x 28563900

[B5] Ballard J. W. O. (2000a). Comparative genomics of mitochondrial DNA in *Drosophila simulans*. *J. Mol. Evol.* 51 64–75. 10.1007/s002390010067 10903373

[B6] Ballard J. W. O. (2000b). Comparative genomics of mitochondrial DNA in members of the *Drosophila melanogaster* subgroup. *J. Mol. Evol.* 51 48–63. 10.1007/s002390010066 10903372

[B7] Barbash D. A. (2010). Genetic testing of the hypothesis that hybrid male lethality results from a failure in dosage compensation. *Genetics* 184 313–316. 10.1534/genetics.109.108100 19841095 PMC2815928

[B8] Barbash D. A. Siino D. F. Tarone A. M. Roote J. (2003). A rapidly evolving Myb-related protein causes species isolation in *Drosophila*. *Proc. Natl. Acad. Sci. U.S.A.* 100 5302–5307. 10.1073/pnas.0836927100 12695567 PMC154340

[B9] Bateson W. (1909). “Heredity and variation in modern lights,” in *Darwin and Modern Science*, ed. Seward A. C. (Cambridge: Cambridge University Press), 85–101.

[B10] Bayes J. J. Malik H. S. (2009). Altered heterochromatin binding by a hybrid sterility protein in *Drosophila* sibling species. *Science* 326 1538–1541. 10.1126/science.1181756 19933102 PMC2987944

[B11] Begun D. J. Holloway A. K. Hillier S. K. L. W. Poh Y. P. Hahn M. W. Nista P. M. (2007). Population genomics: whole-genome analysis of polymorphism and divergence in *Drosophila simulans*. *Public Libr. Sci. Biol.* 5 2534–2559. 10.1371/journal.pbio.0050310 PMC2062478 17988176

[B12] Bengtsson B. O. (1985). “The flow of genes through a genetic barrier,” in *Evolution: Essays in Honour of John Maynard Smith*, eds Greenwood P. J. Harvey P. H. Slatkin M. (Cambridge: Cambridge University Press), 31–42.

[B13] Betran E. Thornton K. Long M. (2002). Retroposed new genes out of the X in *Drosophila*. *Genome Res.* 12 1854–1859. 10.1101/gr.6049 12466289 PMC187566

[B14] Bi Y. Ren X. Yan C. Shao J. Xie D. Zhao Z. (2015). A Genome-wide hybrid incompatibility landscape between *Caenorhabditis briggsae* and *C. nigoni*. *PLoS Genet.* 11:e1004993. 10.1371/journal.pgen.1004993 25692300 PMC4334894

[B15] Brand C. L. Kingan S. B. Garrigan D. (2013). A selective sweep across species boundaries in *Drosophila*. *Mol. Biol. Evol.* 30 2177–2186. 10.1093/molbev/mst123 23827876 PMC3748358

[B16] Brideau N. J. Flores H. A. Wang J. Maheshwari S. Wang X. Barbash D. A. (2006). Two dobzhansky-muller genes interact to cause hybrid lethality in *Drosophila*. *Science* 314 1292–1295. 10.1126/science.1133953 17124320

[B17] Cabot E. L. Davis A. W. Johnson N. A. Wu C.-I. (1994). Genetics of reproductive isolation in the *Drosophila simulans* clade: complex epistasis underlying hybrid male sterility. *Genetics* 137 175–189. 10.1093/genetics/137.1.175 8056308 PMC1205934

[B18] Carvalho A. B. Vaz S. C. Klaczko L. B. (1997). Polymorphism for Y-linked suppressors of sex-ratio in two natural populations of *Drosophila mediopunctata*. *Genetics* 146 891–902. 10.1093/genetics/146.3.891 9215895 PMC1208059

[B19] Cattani M. V. Presgraves D. C. (2009). Genetics and lineage-specific evolution of a lethal hybrid incompatibility between *Drosophila mauritiana* and its sibling species. *Genetics* 181 1545–1555. 10.1534/genetics.108.098392 19189951 PMC2666519

[B20] Chakraborty M. Chang C.-H. Khost D. Vedanayagam J. Adrion J. R. Liao Y. (2021). Evolution of genome structure in the *Drosophila simulans* species complex. *Genome Res.* 31, 380–396. 10.1101/gr.263442.120 33563718 PMC7919458

[B21] Charlesworth B. Coyne J. A. Barton N. H. (1987). The relative rates of evolution of sex-chromosomes and autosomes. *Am. Nat.* 130 113–146. 10.1086/284701

[B22] Charlesworth B. Coyne J. A. Orr H. A. (1993). Meiotic drive and unisexual hybrid sterility - a comment. *Genetics* 133 421–424. 10.1093/genetics/133.2.421 8436280 PMC1205330

[B23] Courret C. Gerard P. R. Ogereau D. Falque M. Moreau L. Montchamp-Moreau C. (2019). X-chromosome meiotic drive in *Drosophila simulans*: a QTL approach reveals the complex polygenic determinism of Paris drive suppression. *Heredity* 122 906–915. 10.1038/s41437-018-0163-1 30518968 PMC6781156

[B24] Coyne J. A. (1984). Genetic basis of male sterility in hybrids between two closely related species of *Drosophila*. *Proc. Natl. Acad. Sci. U.S.A.* 51 4444–4447. 10.1073/pnas.81.14.4444 6589604 PMC345606

[B25] Coyne J. A. (1985). The genetic basis of Haldane’s rule. *Nature* 314 736–738. 10.1038/314736a0 3921852

[B26] Coyne J. A. (1986). Meiotic segregation and male recombination in interspecific hybrids of *Drosophila*. *Genetics* 114 485–494. 10.1093/genetics/114.2.485 3021573 PMC1202952

[B27] Coyne J. A. (1992a). Genetics of sexual isolation in females of the *Drosophila simulans* species complex. *Genet. Res. Camb.* 60 25–31. 10.1017/s0016672300030639 1452013

[B28] Coyne J. A. (1992b). Genetics and speciation. *Nature* 355 511–515. 1741030 10.1038/355511a0

[B29] Coyne J. A. Charlesworth B. (1986). Location of an X-linked factor causing sterility in male hybrids of *Drosophila-simulans* and *Drosophilia-mauritiana*. *Heredity* 57 243–246. 10.1038/hdy.1986.114 3781872

[B30] Coyne J. A. Charlesworth B. (1989). Genetic analysis of X-linked sterility in hybrids between three sibling species of *Drosophila*. *Heredity* 62 97–106. 10.1038/hdy.1989.13 2732092

[B31] Coyne J. A. Charlesworth B. (1997). Genetics of a pheromonal difference affecting sexual isolation between *Drosophila mauritiana* and *D-sechellia*. *Genetics* 145 1015–1030. 10.1093/genetics/145.4.1015 9093854 PMC1207872

[B32] Coyne J. A. Charlesworth B. Orr H. A. (1991). Haldane’s rule revisited. *Evolution* 45 1710–1714. 28564141 10.1111/j.1558-5646.1991.tb02677.x

[B33] Coyne J. A. Kreitman M. (1986). Evolutionary genetics of two sibling species, *Drosophila simulans* and *D. sechellia*. *Evolution* 40 673–691. 10.2307/2408455 28556167

[B34] Coyne J. A. Orr H. A. (1989a). “Two rules of speciation,” in *Speciation and Its Consequences*, eds Otte D. Endler J. (Sunderland, MA: Sinauer Associates), 180–207.

[B35] Coyne J. A. Orr H. A. (1989b). Patterns of speciation in *Drosophila*. *Evolution* 43 362–381. 10.2307/2409213 28568554

[B36] Coyne J. A. Orr H. A. (1993). Further evidence against meiotic-drive models of hybrid sterility. *Evolution* 47 685–687. 10.2307/2410081 28568727

[B37] Coyne J. A. Orr H. A. (1997). “Patterns of speciation in *Drosophila*” revisited. *Evolution* 51 295–303. 10.2307/2410984 28568795

[B38] Coyne J. A. Orr H. A. (2004). *Speciation.* Sunderland, MA: Sinauer.

[B39] Crespi B. J. Nosil P. (2012). Conflictual speciation: species formation via genomic conflict. *Trends Ecol. Evol.* 28 48–57. 10.1016/j.tree.2012.08.015 22995895

[B40] Darwin C. R. (1859). *The Origin of Species.* London: J. Murray.

[B41] David J. McEvey S. F. Solignac M. Tsacas L. (1989). *Drosophila* communities on Mauritius and ecological niche of *D. mauritiana* (Diptera, *Drosophil*idae). *J. Afr. Zool.* 103 107–116.

[B42] Davis A. W. Wu C.-I. (1996). The broom of the sorcerer’s apprentice: the fine structure of a chromosomal region causing reproductive isolation between two sibling species of *Drosophila*. *Genetics* 143 1287–1298. 10.1093/genetics/143.3.1287 8807300 PMC1207397

[B43] Davis B. W. Seabury C. M. Brashear W. A. Li G. Roelke-Parker M. Murphy W. J. (2015). Mechanisms underlying mammalian hybrid sterility in two feline interspecies models. *Mol. Biol. Evol.* 32 2534–2546. 10.1093/molbev/msv124 26006188 PMC4592343

[B44] Delph L. F. Demuth J. P. (2016). Haldane’s rule: genetic bases and their empirical support. *J. Heredity* 107 383–391. 10.1093/jhered/esw026 27233288

[B45] Dobzhansky T. (1936). Studies on hybrid sterility. II. Localization of sterility factors in *Drosophila pseudoobscura* hybrids. *Genetics* 21 113–135. 10.1093/genetics/21.2.113 17246786 PMC1208664

[B46] Dobzhansky T. (1937). *Genetics and the Origin of Species.* New York: Columbia University Press.

[B47] Dobzhansky T. (1940). Speciation as a stage in evolutonary divergence. *Am. Nat.* 74 302–321.

[B48] Dobzhansky T. (1944). On species and races of living and fossil man. *Am. J. Phys. Anthropol.* 2 251–265. 10.1002/ajpa.1330020303

[B49] Ferree P. M. Barbash D. A. (2009). Species-specific heterochromatin prevents mitotic chromosome segregation to cause hybrid lethality in *Drosophila*. *PLoS Biol.* 7:e1000234. 10.1371/journal.pbio.1000234 19859525 PMC2760206

[B50] Ferree P. M. Prasad S. (2012). How can satellite DNA divergence cause reproductive isolation? Let us count the chromosomal ways. *Genet. Res. Int.* 2012:430136. 10.1155/2012/430136 PMC3335601 22567387

[B51] Fraisse C. Sachdeva H. (2020). The rates of introgression and barriers to genetic exchange between hybridizing species: sex chromosomes vs autosomes. *Genetics* 217:iyaa025. 10.1093/genetics/iyaa025 PMC8045713 33724409

[B52] Frank S. A. (1991). Haldane’s rule: a defense of the meiotic drive theory. *Evolution* 45 1714–1716. 10.2307/2409793 28564136

[B53] Frazee S. R. Masly J. P. (2015). Multiple sexual selection pressures drive the rapid evolution of complex morphology in a male secondary genital structure. *Ecol. Evol.* 5 4437–4450. 10.1002/ece3.1721 26664690 PMC4667835

[B54] Gallach M. (2014). Recurrent turnover of chromosome-specific satellites in *Drosophila*. *Genome Biol. Evol.* 6 1279–1286. 10.1093/gbe/evu104 24846631 PMC4079201

[B55] Garrigan D. Kingan S. B. Geneva A. J. Andolfatto P. Clark A. G. Thornton K. R. (2012). Genome sequencing reveals complex speciation in the *Drosophila simulans* clade. *Genome Res.* 22 1499–1511. 10.1101/gr.130922.111 22534282 PMC3409263

[B56] Garrigan D. Kingan S. B. Geneva A. J. Vedanayagam J. P. Presgraves D. C. (2014). Genome diversity and divergence in *Drosophila mauritiana*: multiple signatures of faster X evolution. *Genome Biol. Evol.* 6 2444–2458. 10.1093/gbe/evu198 25193308 PMC4202334

[B57] Gelbart M. E. Kuroda M. I. (2009). *Drosophila* dosage compensation: a complex voyage to the X chromosome. *Development* 136 1399–1410. 10.1242/dev.029645 19363150 PMC2674252

[B58] Geneva A. J. Muirhead C. Kingan S. B. Garrigan D. (2016). A new method to scan genomes for introgression in a secondary contact model. *PLoS One* 10:e0118621. 10.1371/journal.pone.0118621 25874895 PMC4396994

[B59] Gingell L. F. McLean J. R. (2020). A protamine knockdown mimics the function of Sd in *Drosophila melanogaster*. *G3* 10 2111–2115. 10.1534/g3.120.401307 32321837 PMC7263674

[B60] Good J. M. Dean M. D. Nachman M. W. (2008). A complex genetic basis to X-Linked hybrid male sterility between two species of house mice. *Genetics* 179 2213–2228. 10.1534/genetics.107.085340 18689897 PMC2516092

[B61] Haldane J. B. S. (1922). Sex ratio and unisexual sterility in animal hybrids. *J. Genet.* 12 101–109. 10.1007/bf02983075 27974520

[B62] Hall D. W. (2004). Meiotic drive and sex chromosome cycling. *Evolution* 58 925–931. 10.1554/03-440 15212373

[B63] Hamilton W. D. (1967). Extraordinary sex ratios. *Science* 156 477–488. 10.1126/science.156.3774.477 6021675

[B64] Han M. V. Hahn M. W. (2012). Inferring the history of interchromosomal gene transposition in *Drosophila* using n-dimensional parsimony. *Genetics* 190 813–825. 10.1534/genetics.111.135947 22095076 PMC3276645

[B65] Helleu Q. Courret C. Ogereau D. Burnham K. L. Chaminade N. Chakir M. (2019). Sex-ratio meiotic drive shapes the evolution of the Y chromosome in *Drosophila* simulans. *Mol. Biol. Evol.* 36 2668–2681. 10.1093/molbev/msz160 31290972

[B66] Helleu Q. Gerard P. R. Dubruille R. Ogereau D. Prud’homme N. Loppin B. (2016). Rapid evolution of a Y-chromosome heterochromatin protein underlies sex chromosome meiotic drive. *Proc. Natl. Acad. Sci. U.S.A.* 113 4110–4115. 10.1073/pnas.1519332113 26979956 PMC4839453

[B67] Hense W. Baines J. F. Parsch J. (2007). X chromosome inactivation during *Drosophila* spermatogenesis. *Public Libr. Sci. Biol.* 5 2288–2295. 10.1371/journal.pbio.0050273 PMC2001211 17927450

[B68] Hollocher H. Wu C.-I. (1996). The genetics of reproductive isolation in the *Drosophila simulans* clade: X *vs*. autosomal effects and male *vs*. female effects. *Genetics* 143 1243–1255. 10.1093/genetics/143.3.1243 8807297 PMC1207394

[B69] Hsieh T. Brutlag D. (1979). Sequence and sequence variation within the 1.688 g/cm3 satellite DNA of *Drosophila melanogaster*. *J. Mol. Biol.* 135 465–481. 10.1016/0022-2836(79)90447-9 231676

[B70] Hurst L. D. Pomiankowski A. (1991). Causes of sex ratio bias may account for unisexual sterility in hybrids: a new explanation of Haldane’s rule and related phenomena. *Genetics* 128 841–858. 10.1093/genetics/128.4.841 1916248 PMC1204557

[B71] Hutter P. Ashburner M. (1987). Genetic rescue of inviable hybrids between *Drosophila melanogaster* and its sibling species. *Nature* 327 331–333. 10.1038/327331a0 3108667

[B72] Jaenike J. (2001). Sex chromosome meiotic drive. *Annu. Rev. Ecol. Syst.* 32 25–49.

[B73] Jagannathan M. Warsinger-Pepe N. Watase G. J. Yamashita Y. (2017). Comparative analysis of satellite DNA in the *Drosophila melanogaster* species complex. *G3* 3 693–704. 10.1534/g3.116.035352 28007840 PMC5295612

[B74] Johnson N. (2010). Hybrid incompatibility genes: remnants of a genomic battlefield? *Trends Genet.* 26 317–325. 10.1016/j.tig.2010.04.005 20621759

[B75] Johnson N. A. Hollocher H. Noonburg E. Wu C.-I. (1993). The effects of interspecific Y chromosome replacements on hybrid sterility within the *Drosophila simulans* clade. *Genetics* 135 443–453. 10.1093/genetics/135.2.443 8244006 PMC1205647

[B76] Johnson N. A. Perez D. E. Cabot E. L. Hollocher H. Wu C.-I. (1992). A test of reciprocal X-Y interactions as a cause of hybrid sterility in *Drosophila*. *Nature* 358 751–753. 10.1038/358751a0 1508270

[B77] Johnson N. A. Porter A. H. (2000). Rapid speciation via parallel, directional selection on regulatory genetic pathways. *J. Theor. Biol.* 205 527–542. 10.1006/jtbi.2000.2070 10931750

[B78] Johnson N. A. Wu C.-I. (1992). An empirical test of the meiotic drive models of hybrid sterility: sex ratio data from hybrids between *Drosophila simulans* and *Drosophila sechellia*. *Genetics* 130 507–511. 10.1093/genetics/130.3.507 1551573 PMC1204868

[B79] Kanippayoor R. L. Alpern J. H. M. Moehring A. J. (2020). A common suite of cellular abnormalities and spermatogenetic errors in sterile hybrid males in *Drosophila*. *Proc. R. Soc. B Biol. Sci.* 287:20192291. 10.1098/rspb.2019.2291 31964309 PMC7015338

[B80] Kliman R. M. Andolfatto P. Coyne J. A. Depaulis F. Kreitman M. Berry A. J. (2000). The population genetics of the origin and divergence of the *Drosophila simulans* complex species. *Genetics* 156 1913–1931. 11102384 10.1093/genetics/156.4.1913 PMC1461354

[B81] Kruger A. N. Brogley M. A. Huizinga J. L. Kidd J. M. de Rooij D. G. Hu Y. C. (2019). A neofunctionalized X-linked ampliconic gene family is essential for male fertility and equal sex ratio in mice. *Curr. Biol.* 29 3699.e5–3706.e5. 31630956 10.1016/j.cub.2019.08.057 PMC7012382

[B82] Kuhn G. C. Kuttler H. Moreira-Filho O. Heslop-Harrison J. S. (2012). The 1.688 repetitive DNA of *Drosophila*: concerted evolution at different genomic scales and association with genes. *Mol. Biol. Evol.* 29 7–11. 10.1093/molbev/msr173 21712468

[B83] Kulathinal R. J. Singh R. S. (1998). Cytological characterization of premeiotic versus postmeiotic defects producing hybrid male sterility among sibling species of the *Drosophila melanogaster* complex. *Evolution* 52 1067–1079. 10.2307/2411237 28565214

[B84] Lachaise D. Cariou M.-L. David J. R. Lemeunier F. Tsacas L. Ashburner M. (1988). Historical biogeography of the *Drosophila melanogaster* species subgroup. *Evol. Biol.* 22 159–225. 10.1007/978-1-4613-0931-4_4

[B85] Lachaise D. David J. R. Lemeunier F. Tsacas L. Ashburner M. (1986). The reproductive relationships of *Drosophila sechellia* with *D. mauritiana*, *D. simulans*, and *D. melanogaster* from the Afrotropical region. *Evolution* 1986 262–271. 10.1111/j.1558-5646.1986.tb00468.x 28556049

[B86] Landeen E. L. Muirhead C. Wright L. Meiklejohn C. D. Presgraves D. C. (2016). Sex chromosome-wide transcriptional suppression and compensatory *cis*-regulatory evolution mediate gene expression in the *Drosophila* male germline. *PLoS Biol.* 14:e1002499. 10.1371/journal.pbio.1002499 27404402 PMC4942098

[B87] Larracuente A. M. (2014). The organization and evolution of the Responder satellite in species of the *Drosophila melanogaster* group: dynamic evolution of a target of meiotic drive. *BMC Evol. Biol.* 14:233. 10.1186/s12862-014-0233-9 25424548 PMC4280042

[B88] Laurie C. C. (1997). The weaker sex is heterogametic: 75 years of Haldane’s rule. *Genetics* 147 937–951. 10.1093/genetics/147.3.937 9383043 PMC1208269

[B89] Levine M. T. Holloway A. K. Arshad U. Begun D. J. (2007). Pervasive and largely lineage-specific adaptive protein evolution in the dosage compensation complex of *Drosophila melanogaster*. *Genetics* 177 1959–1962. 10.1534/genetics.107.079459 18039888 PMC2147993

[B90] Lienard M. A. Araripe L. O. Hartl D. L. (2016). Neighboring genes for DNA-binding proteins rescue male sterility in *Drosophila* hybrids. *Proc. Natl. Acad. Sci. U.S.A.* 113 E4200–E4207. 27357670 10.1073/pnas.1608337113 PMC4961176

[B91] Lin C. J. Hu F. Dubruille R. Vedanayagam J. Wen J. Smibert P. (2018). The hpRNA/RNAi pathway is essential to resolve intragenomic conflict in the *Drosophila* male germline. *Dev. Cell.* 46 316.e5–326.e5. 30086302 10.1016/j.devcel.2018.07.004 PMC6114144

[B92] Lindsley D. L. Roote J. Kennison J. A. (2013). Anent the genomics of spermatogenesis in *Drosophila melanogaster*. *PLoS One* 8:e55915. 10.1371/journal.pone.0055915 23409089 PMC3567030

[B93] Lindsley D. L. Tokuyasu K. T. (1980). “Spermatogenesis,” in *The Genetics and Biology of Drosophila*, eds Ashburner M. Wright T. R. F. (New York: Academic Press), 226–294.

[B94] Lu X. Shapiro J. A. Ting C.-T. Li Y. Li C. Xu J. (2010). Genome-wide misexpression of X-linked versus autosomal genes associated with hybrid male sterility. *Genome Res.* 20 1097–1102. 10.1101/gr.076620.108 20511493 PMC2909572

[B95] Lynch M. Force A. G. (2000). The origin of interspecific genomic incompatibility via gene duplication. *Am. Nat.* 156 590–605. 10.2307/3079065 29592543

[B96] Mack K. L. Nachman M. W. (2016). Gene regulation and speciation. *Trends Genet.* 33 68–80. 10.1016/j.tig.2016.11.003 27914620 PMC5182078

[B97] Mallet J. (2005). Hybridization as an invasion of the genome. *Trends Ecol. Evol.* 20 229–237. 10.1016/j.tree.2005.02.010 16701374

[B98] Mallet J. (2006). What does *Drosophila* genetics tell us about speciation? *Trends Ecol. Evol.* 21 386–393. 10.1016/j.tree.2006.05.004 16765478

[B99] Mallet J. Besansky N. Hahn M. W. (2016). How reticulated are species? *BioEssays* 38 140–149. 10.1002/bies.201500149 26709836 PMC4813508

[B100] Masly J. P. Jones C. D. Noor M. A. F. Locke J. Orr H. A. (2006). Gene transposition as a novel cause of hybrid male sterility. *Science* 313 1448–1450. 10.1126/science.1128721 16960009

[B101] Masly J. P. Presgraves D. C. (2007). High-resolution genome-wide dissection of the two rules of speciation in *Drosophila*. *Public Libr. Sci. Biol.* 5 1890–1898. 10.1371/journal.pbio.0050243 PMC1971125 17850182

[B102] Matute D. R. Ayroles J. F. (2014). Hybridization occurs between *Drosophila simulans* and *D. sechellia* in the *Seychelles archipelago*. *J. Evol. Biol.* 27 1057–1068. 10.1111/jeb.12391 24773151

[B103] McDermott S. R. Kliman R. M. (2008). Estimation of isolation times of the island species in the *Drosophila simulans* complex from multilocus sequence data. *PLoS One* 3:e2442. 10.1371/journal.pone.0002442 18560591 PMC2413010

[B104] Meany M. K. Conner W. R. Richter S. V. Bailey J. A. Turelli M. Cooper B. S. (2019). Loss of cytoplasmic incompatibility and minimal fecundity effects explain relatively low *Wolbachia* frequencies in *Drosophila mauritiana*. *Evolution* 73 1278–1295. 10.1111/evo.13745 31001816 PMC6554066

[B105] Meiklejohn C. D. Landeen E. L. Cook J. M. Kingan S. B. Presgraves D. C. (2011). Sex chromosome-specific regulation in the *Drosophila* male germline but little evidence for chromosomal dosage compensation or meiotic inactivation. *PLoS Biol.* 9:e1001126. 10.1371/journal.pbio.1001126 21857805 PMC3156688

[B106] Meiklejohn C. D. Landeen E. L. Gordon K. E. Rzatkiewicz T. Kingan S. B. Geneva A. J. (2018). Gene flow mediates the role of sex chromosome meiotic drive during complex speciation. *eLife* 7:e35468. 10.7554/eLife.35468 PMC6292695 30543325

[B107] Meiklejohn C. D. Parsch J. Ranz J. M. Hartl D. L. (2003). Rapid evolution of male-biased gene expression in *Drosophila*. *Proc. Natl. Acad. Sci. U.S.A.* 100 9894–9899. 10.1073/pnas.1630690100 12907700 PMC188344

[B108] Meiklejohn C. D. Presgraves D. C. (2012). Little evidence for demasculinization of the *Drosophila X* chromosome among genes expressed in the male germline. *Genome Biol. Evol.* 4 895–904. 10.1093/gbe/evs077 PMC3490416 22975718

[B109] Meiklejohn C. D. Tao Y. (2010). Genetic conflict and sex chromosome evolution. *Trends Ecol. Evol.* 25 215–223. 10.1016/j.tree.2009.10.005 19931208 PMC2843792

[B110] Meisel R. P. Connallon T. (2013). The faster-X effect: integrating theory and data. *Trends Genet.* 29 537–544. 10.1016/j.tig.2013.05.009 23790324 PMC3755111

[B111] Meisel R. P. Han M. V. Hahn M. W. (2009). A complex suite of forces drives gene traffic from *Drosophila* X chromosomes. *Genome Biol. Evol.* 1 176–188. 10.1093/gbe/evp018 20333188 PMC2817413

[B112] Meisel R. P. Malone J. H. Clark A. G. (2012). Faster-X evolution of gene expression in *Drosophila*. *PLoS Genet.* 8:e1003013. 10.1371/journal.pgen.1003013 23071459 PMC3469423

[B113] Miller G. T. Pitnick S. (2002). Sperm-female coevolution in *Drosophila*. *Science* 298 1230–1233. 10.1126/science.1076968 12424377

[B114] Mills W. K. Lee Y. C. G. Kochendoerfer A. M. Dunleavy E. M. Karpen G. H. (2019). RNA from a simple-tandem repeat is required for sperm maturation and male fertility in *Drosophila melanogaster*. *eLife* 8:e48940. 10.7554/eLife.48940 PMC6879302 31687931

[B115] Moehring A. J. Llopart A. Elwyn S. Coyne J. A. Mackay T. F. (2006a). The genetic basis of postzygotic reproductive isolation between *Drosophila santomea* and *D. yakuba* due to hybrid male sterility. *Genetics* 173 225–233. 10.1534/genetics.105.052985 16510788 PMC1461443

[B116] Moehring A. J. Teeter K. C. Noor M. A. F. (2006b). Genome-wide patterns of expression in *Drosophila* pure species and hybrid males. II. Examination of multiple-species hybridizations, platforms, and life cycle stages. *Mol. Biol. Evol.* 24 137–145. 10.1093/molbev/msl142 17032727

[B117] Moyle L. C. Muir C. D. Han M. V. Hahn M. W. (2010). The contribution of gene movement to the two rules of speciation. *Evolution* 64 1541–1557. 10.1111/j.1558-5646.2010.00990.x 20298429

[B118] Muirhead C. Presgraves D. C. (2016). Hybrid incompatibilities, local adaptation, and the genomic distribution of natural introgression between species. *Am. Nat.* 187 249–261. 10.1086/684583 26807751

[B119] Muller H. J. (1940). “Bearing of the *Drosophila* work on systematics,” in *The New Systematics*, ed. Huxley J. S. (Oxford: Clarendon Press), 185–268.

[B120] Muller H. J. (1942). Isolating mechanisms, evolution, and temperature. *Biol. Symp.* 6 71–125.

[B121] Muller H. J. Pontecorvo G. (1940). Recombinants between *Drosophila* species, the F_1_ hybrids of which are sterile. *Nature* 146:199. 10.1038/146199b0

[B122] Navascues M. Legrand D. Campagne C. Cariou M. L. Depaulis F. (2014). Distinguishing migration from isolation using genes with intragenic recombination: detecting introgression in the *Drosophila simulans* species complex. *BMC Evol. Biol.* 14:89. 10.1186/1471-2148-14-89 24762206 PMC4022370

[B123] Naveira H. Fontdevila A. (1985). The evolutionary history of *Drosophila buzzattii*. IX. High frequencies of chromosome rearrangements induced by introgressive hybridization. *Chromosoma* 91 87–94. 10.1007/bf00294050 3987443

[B124] Naveira H. Fontdevila A. (1986). The evolutionary history of *Drosophila buzzatii*. XII. The genetic basis of sterility in hybrids between *D. buzzatii* and its sibling *D. serido* from Argentina. *Genetics* 114 841–857. 10.1093/genetics/114.3.841 17246354 PMC1203016

[B125] Naveira H. Fontdevila A. (1991). The evolutionary history of *Drosophila buzzatii*. XXI. Cumulative action of multiple sterility factors on spermatogenesis in hybrids of *D. buzzatii* and *D. koepferae*. *Heredity* 67 57–72. 10.1038/hdy.1991.65 1917552

[B126] Naveira H. F. (1992). Location of X-linked polygenic effects causing sterility in male hybrids of *Drosophila simulans* and *D. mauritiana*. *Heredity* 68 211–217. 10.1038/hdy.1992.34 1559838

[B127] Naveira H. F. (2003). On the relative roles of faster-X evolution and dominance in the establishment of intrinsic postzygotic isolating barriers. *Genetica* 118 41–50. 12733663 10.1023/a:1022978222021

[B128] Naveira H. F. Maside X. R. (1998). “The genetics of hybrid male sterility in *Drosophila*,” in *Endless Forms*, eds Howard D. J. Berlocher S. H. (Oxford: Oxford University Press), 330–338.

[B129] Nolte V. Pandey R. V. Kofler R. Schlotterer C. (2013). Genome-wide patterns of natural variation reveal strong selective sweeps and ongoing genomic conflict in *Drosophila mauritiana*. *Genome Res.* 23 99–110. 10.1101/gr.139873.112 23051690 PMC3530687

[B130] Nunes M. D. Wengel P. O. Kreissl M. Schlotterer C. (2010). Multiple hybridization events between *Drosophila simulans* and *Drosophila mauritiana* are supported by mtDNA introgression. *Mol. Ecol.* 19 4695–4707. 10.1111/j.1365-294x.2010.04838.x 20958812 PMC3035818

[B131] Orr H. A. (1989). Does postzygotic isolation result from improper dosage compensation? *Genetics* 122 891–894. 10.1093/genetics/122.4.891 2503427 PMC1203763

[B132] Orr H. A. (1992). Mapping and characterization of a speciation gene in *Drosophila*. *Genet. Res.* 59 73–80. 10.1017/s0016672300030275 1628819

[B133] Orr H. A. (1996). Dobzhansky, Bateson, and the genetics of speciation. *Genetics* 144 1331–1335. 10.1093/genetics/144.4.1331 8978022 PMC1207686

[B134] Orr H. A. (1997). Haldane’s rule. *Annu. Rev. Ecol. Syst.* 28 195–218.

[B135] Orr H. A. Betancourt A. J. (2001). Haldane’s sieve and adaptation from the standing genetic variation. *Genetics* 157 875–884. 10.1093/genetics/157.2.875 11157004 PMC1461537

[B136] Orr H. A. Irving S. (2005). Segregation distortion in hybrids between the bogota and USA subspecies of *Drosophila pseudoobscura*. *Genetics* 169 671–682. 10.1534/genetics.104.033274 15654115 PMC1449097

[B137] Palopoli M. F. Wu C.-I. (1994). Genetics of hybrid male sterility between *Drosophila* sibling species: a complex web of epistasis is revealed in interspecific studies. *Genetics* 138 329–341. 10.1093/genetics/138.2.329 7828817 PMC1206152

[B138] Perez D. E. Wu C.-I. (1995). Further characterization of the *Odysseus* locus of hybrid sterility in *Drosophila*: one gene is not enough. *Genetics* 140 201–206. 10.1093/genetics/140.1.201 7635285 PMC1206547

[B139] Perez D. E. Wu C.-I. Johnson N. A. Wu M.-L. (1993). Genetics of reproductive isolation in the *Drosophila simulans* clade: DNA-marker assisted mapping and characterization of a hybrid-male sterility gene, *Odysseus* (Ods). *Genetics* 134 261–275. 10.1093/genetics/134.1.261 8514135 PMC1205429

[B140] Phadnis N. (2011). Genetic architecture of male sterility and segregation distortion in *Drosophila pseudoobscura* Bogota-USA hybrids. *Genetics* 189 1001–1009. 10.1534/genetics.111.132324 21900263 PMC3213365

[B141] Phadnis N. Baker E. C. P. Cooper J. C. Frizzell K. A. Hsieh E. de la Cruz A. F. A. (2015). An essential cell cycle regulation gene causes hybrid inviability in *Drosophila*. *Science* 350 1552–1555. 10.1126/science.aac7504 26680200 PMC4703311

[B142] Phadnis N. Orr H. A. (2008). A single gene causes both male sterility and segregation distortion in *Drosophila* hybrids. *Science* 323 376–379. 10.1126/science.1163934 19074311 PMC2628965

[B143] Presgraves D. C. (2002). Patterns of postzygotic isolation in Lepidoptera. *Evolution* 56 1168–1183. 10.1554/0014-3820(2002)056[1168:popiil]2.0.co;2 12144018

[B144] Presgraves D. C. (2008a). Sex chromosomes and speciation in *Drosophila*. *Trends Genet.* 24 336–343. 10.1016/j.tig.2008.04.007 18514967 PMC2819171

[B145] Presgraves D. C. (2008b). “Drive and sperm: evolution and genetics of male meiotic drive,” in *Sperm Biology: An Evolutionary Perspective*, eds Birkhead T. R. Hosken D. J. Pitnick S. (Amsterdam: Elsevier Press).

[B146] Presgraves D. C. (2010). The molecular evolutionary basis of species formation. *Nat. Rev. Genet.* 11 175–180. 10.1038/nrg2718 20051985

[B147] Presgraves D. C. (2018). Evaluating genomic signatures of the large X-effect during complex speciation. *Mol. Ecol.* 27 3822–3830. 10.1111/mec.14777 29940087 PMC6705125

[B148] Presgraves D. C. Balagopalan L. Abmayr S. M. Orr H. A. (2003). Adaptive evolution drives divergence of a hybrid inviability gene between two species of *Drosophila*. *Nature* 423 715–719. 10.1038/nature01679 12802326

[B149] Price C. S. C. (1997). Conspecific sperm precedence in *Drosophila*. *Nature* 388 663–666. 10.1038/41753 9262398

[B150] Price T. D. Bouvier M. M. (2002). The evolution of F1 postzygotic incompatibilities in birds. *Evolution* 56 2083–2089. 10.1554/0014-3820(2002)056[2083:teofpi]2.0.co;2 12449494

[B151] Rabosky D. L. Matute D. R. (2013). Macroevolutionary speciation rates are decoupled from the evolution of intrinsic reproductive isolation in *Drosophila* and birds. *Proc. Natl. Acad. Sci. U.S.A.* 110 15354–15359. 10.1073/pnas.1305529110 24003144 PMC3780891

[B152] Rathje C. C. Johnson E. E. P. Drage D. Patinioti C. G. Silvestri G. Affara N. A. (2019). Differential sperm motility mediates the sex ratio drive shaping mouse sex chromosome evolution. *Curr. Biol.* 29 3692–3698. 10.1016/j.cub.2019.09.031 31630954 PMC6839398

[B153] Rathke C. Baarends W. M. Jayaramaiah-Raja S. Bartkuhn M. Renkawitz R. Renkawitz-Pohl R. (2007). Transition from a nucleosome-based to a protamine-based chromatin configuration during spermiogenesis in *Drosophila*. *J. Cell Sci.* 120 1689–1700. 10.1242/jcs.004663 17452629

[B154] R’Kha S. Capy P. David J. R. (1991). Host-plant specialization in the *Drosophila melanogaster* species complex: a physiological, behavioral, and genetical analysis. *Proc. Natl. Acad. Sci. U.S.A.* 88 1835–1839. 10.1073/pnas.88.5.1835 1900368 PMC51120

[B155] Rockman M. V. (2012). The QTN program and the alleles that matter for evolution: all that’s gold does not glitter. *Evolution* 66 1–17. 10.1111/j.1558-5646.2011.01486.x 22220860 PMC3386609

[B156] Rodriguez M. A. Vermaak D. Bayes J. J. Malik H. S. (2007). Species-specific positive selection of the male-specific lethal complex that participates in dosage compensation in *Drosophila*. *Proc. Natl. Acad. Sci. U.S.A.* 104 15412–15417. 10.1073/pnas.0707445104 17878295 PMC2000485

[B157] Rose M. Doolittle W. F. (1983). Molecular biological mechanisms of speciation. *Science* 220 157–162. 10.1126/science.220.4593.157 17795801

[B158] Satta Y. Takahata N. (1990). Evolution of *Drosophila* mitochondrial DNA and the history of the melanogaster subgroup. *Proc. Natl. Acad. Sci. U.S.A.* 87 9558–9562. 10.1073/pnas.87.24.9558 2124697 PMC55211

[B159] Satyaki P. R. Cuykendall T. N. Wei K. H. Brideau N. J. Kwak H. Aruna S. (2014). The Hmr and Lhr hybrid incompatibility genes suppress a broad range of heterochromatic repeats. *PLoS Genet.* 10:e1004240. 10.1371/journal.pgen.1004240 24651406 PMC3961192

[B160] Sawamura K. (2000). Genetics of hybrid inviability and sterility in *Drosophila*: the *Drosophila melanogaster*-*Drosophila simulans* case. *Plant Species Biol.* 15 237–247. 10.1046/j.1442-1984.2000.00043.x

[B161] Sawamura K. (2012). Chromatin evolution and molecular drive in speciation. *Int. J. Evol. Biol.* 2012:301894. 10.1155/2012/301894 PMC3235502 22191063

[B162] Sawamura K. Davis A. W. Wu C.-I. (2000). Genetic analysis of speciation by means of introgression into *Drosophila melanogaster*. *Proc. Natl. Acad. Sci. U.S.A.* 97 2652–2655. 10.1073/pnas.050558597 10706624 PMC15984

[B163] Sawamura K. Yamamoto M. T. (1997). Characterization of a reproductive isolation gene, Zygotic hybrid rescue, of *Drosophila melanogaster* by using minichromosomes. *Heredity* 79 97–103. 10.1038/hdy.1997.127

[B164] Schilthuizen M. Giesbers M. C. W. G. Beukeboom L. W. (2011). Haldane’s rule in the 21st century. *Heredity* 107 95–102. 21224879 10.1038/hdy.2010.170 PMC3178397

[B165] Schrider D. R. Ayroles J. Matute D. R. Kern A. D. (2018). Supervised machine learning reveals introgressed loci in the genomes of *Drosophila simulans* and *D. sechellia*. *PLoS Genet.* 14:e1007341. 10.1371/journal.pgen.1007341 29684059 PMC5933812

[B166] Seehausen O. Butlin R. K. Keller I. Wagner C. E. Boughman J. W. Hohenlohe P. A. (2014). Genomics and the origin of species. *Nat. Rev. Genet.* 15 176–192. 24535286 10.1038/nrg3644

[B167] Solignac M. Monnerot M. (1986). Race formation, speciation, and introgression within *Drosophila simulans*, *D. mauritiana*, and *D. sechellia* inferred from mitonchondrial DNA analysis. *Evolution* 40 531–539. 10.1111/j.1558-5646.1986.tb00505.x 28556334

[B168] Solignac M. Monnerot M. Mounolou J.-C. (1986). Mitochondrial DNA evolution in the melanogaster species subgroup of *Drosophila*. *J. Mol. Evol.* 23 31–40. 3009834 10.1007/BF02100996

[B169] Sproul J. S. Khost D. E. Eickbush D. G. Negm S. Wei X. Wong I. (2020). Dynamic evolution of euchromatic satellites on the X Chromosome in *Drosophila melanogaster* and the simulans Clade. *Mol. Biol. Evol.* 37 2241–2256. 10.1093/molbev/msaa078 32191304 PMC7403614

[B170] Stern C. (1936). Interspecific sterility. *Am. Nat.* 70 123–142. 10.1086/280648

[B171] Sturtevant A. H. (1919). A new species closely resembling *Drosophila melanogaster*. *Psyche* 26 153–155. 10.1155/1919/97402

[B172] Sturtevant A. H. (1920). Genetic studies on *Drosophila simulans*. I. Introduction. Hybrids with *Drosophila melanogaster*. *Genetics* 5 488–500. 10.1093/genetics/5.5.488 17245951 PMC1200491

[B173] Swanson W. J. Clark A. G. Waldrip-Dail H. M. Wolfner M. F. Aquadro C. F. (2001). Evolutionary EST analysis identifies rapidly evolving male reproductive proteins in *Drosophila*. *Proc. Natl. Acad. Sci. U.S.A.* 98 7375–7379. 10.1073/pnas.131568198 11404480 PMC34676

[B174] Tang S. Presgraves D. C. (2009). Evolution of the *Drosophila* nuclear pore complex results in multiple hybrid incompatibilities. *Science* 323 779–782. 10.1126/science.1169123 19197064 PMC2826207

[B175] Tao Y. Araripe L. Kingan S. B. Ke Y. Xiao H. Hartl D. L. (2007a). A sex-ratio meiotic drive system in *Drosophila simulans*. II: an X-linked distorter. *Public Libr. Sci. Biol.* 5:e293. 10.1371/journal.pbio.0050293 17988173 PMC2062476

[B176] Tao Y. Chen S. Hartl D. L. Laurie C. C. (2003a). Genetic dissection of hybrid incompatibilities between *Drosophila simulans* and *D. mauritiana*. I. Differential accumulation of hybrid male sterility effects on the X and autosomes. *Genetics* 164 1383–1397. 10.1093/genetics/164.4.1383 12930747 PMC1462656

[B177] Tao Y. Masly J. P. Araripe L. Ke Y. Hartl D. L. (2007b). A sex-ratio meiotic drive system in *Drosophila simulan*s. I: an autosomal suppressor. *Public Libr. Sci. Biol.* 5:e292. 10.1371/journal.pbio.0050292 17988172 PMC2062475

[B178] Tao Y. Hartl D. L. (2003). Genetic dissection of hybrid incompatibilities between *Drosophila simulans* and *D. mauritiana*. III. Heterogeneous accumulation of hybrid incompatibilities, degree of dominance, and implications for Haldane’s rule. *Evolution* 57 2580–2589. 10.1554/03-094 14686533

[B179] Tao Y. Hartl D. L. Laurie C. C. (2001). Sex-ratio segregation distortion associated with reproductive isolation in *Drosophila*. *Proc. Natl. Acad. Sci. U.S.A.* 98 13183–13188. 10.1073/pnas.231478798 11687638 PMC60845

[B180] Tao Y. Zeng Z. B. Hartl D. L. Laurie C. C. (2003b). Genetic dissection of hybrid incompatibilities between *Drosophila simulans* and *D. mauritiana*. II. Mapping hybrid male sterility loci on the third chromosome. *Genetics* 164 1399–1418. 10.1093/genetics/164.4.1399 12930748 PMC1462659

[B181] Ting C.-T. Tsaur S.-C. Wu M.-L. Wu C.-I. (1998). A rapidly evolving homeobox at the site of a hybrid sterility gene. *Science* 282 1501–1504. 10.1126/science.282.5393.1501 9822383

[B182] True J. R. Mercer J. M. Laurie C. C. (1996b). Differences in crossover frequency and distribution among three sibling species of *Drosophila*. *Genetics* 142 507–523. 10.1093/genetics/142.2.507 8852849 PMC1206984

[B183] True J. R. Weir B. S. Laurie C. C. (1996a). A genome-wide survey of hybrid incompatibility factors by the introgression of marked segments of *Drosophila mauritiana* chromosomes into *Drosophila simulans*. *Genetics* 142 819–837. 10.1093/genetics/142.3.819 8849890 PMC1207021

[B184] Tsacas L. Bächli G. (1981). *Drosophila sechellia*, n.sp., huitieme espece du sous-goupe melanogaster des Iles Sechelles [Diptera, Drosophilidae]. *Rev. Franc. D’entomol. Nouvelle Ser.* 3 146–150.

[B185] Tsacas L. David J. R. (1974). *Drosophila mauritiana* n.sp. du groupe melanogaster de l’Ile Maurice. *Bull. Soc. Entomol. France* 79 42–46.

[B186] Turelli M. Orr H. A. (1995). The dominance theory of Haldane’s rule. *Genetics* 140 389–402. 10.1093/genetics/140.1.389 7635302 PMC1206564

[B187] Turelli M. Orr H. A. (2000). Dominance, epistasis and the genetics of postzygotic isolation. *Genetics* 154 1663–1679. 10.1093/genetics/154.4.1663 10747061 PMC1461023

[B188] Vibranovski M. D. Lopes H. F. Karr T. L. Long M. (2009). Stage-specific expression profiling of *Drosophila* spermatogenesis suggests that meiotic sex chromosome inactivation drives genomic relocation of testis-expressed genes. *PLoS Genet.* 5:e1000731. 10.1371/journal.pgen.1000731 19936020 PMC2770318

[B189] Watanabe T. K. (1979). A gene that rescues the lethal hybrids between *Drosophila melanogaster* and *D. simulans*. *Jpn. J. Genet.* 54 325–331. 10.1266/jjg.54.325

[B190] Werth C. R. Windham M. D. (1991). A model for divergent, allopatric speciation of polyploid pteridophytes resulting from silencing of duplicate-gene expression. *Am. Nat.* 137 515–526. 10.1086/285180

[B191] Wu C.-I. (1992). A note on Haldane’s rule: hybrid inviability versus hybrid sterility. *Evolution* 46 1584–1587. 10.2307/2409965 28569002

[B192] Wu C.-I. (2001). The genic view of the process of speciation. *J. Evol. Biol.* 14 851–865. 10.1046/j.1420-9101.2001.00335.x

[B193] Wu C.-I. Davis A. W. (1993). Evolution of postmating reproductive isolation: the composite nature of Haldane’s rule and its genetic bases. *Am. Nat.* 142 187–212. 10.1086/285534 19425975

[B194] Wu C.-I. Hollocher H. (1998). “Subtle is Nature: the genetics of species differentiation and speciation,” in *Endless Forms*, eds Howard D. J. Berlocher S. H. (Oxford: Oxford University Press), 339–351.

[B195] Wu C.-I. Johnson N. A. Palopoli M. F. (1996). Haldane’s rule and its legacy: why are there so many sterile males? *Trends Ecol. Evol.* 11 281–284. 10.1016/0169-5347(96)10033-1 21237844

[B196] Wu C.-I. Lyttle T. W. Wu M.-L. Lin G. F. (1988). Association between DNA satellite sequences and the responder of segregation distortion in *D. melanogaster*. *Cell* 54 179–189. 10.1016/0092-8674(88)90550-8 2839299

[B197] Wu C.-I. Palopoli M. F. (1994). Genetics of postmating reproductive isolation in animals. *Ann. Rev. Genet.* 27 283–308. 10.1146/annurev.ge.28.120194.001435 7893128

[B198] Yukilevich R. (2012). Asymmetrical patterns of speciation uniquely support reinforcement in *Drosophila*. *Evolution* 66 1430–1446. 10.1111/j.1558-5646.2011.01534.x 22519782

[B199] Zeng L.-W. Singh R. S. (1993). The genetic basis of Haldane’s rule and the nature of asymmetric hybrid male sterility among *Drosophila simulans*, *Drosophila mauritiana* and *Drosophila sechellia*. *Genetics* 134 251–260. 10.1093/genetics/134.1.251 8514134 PMC1205428

[B200] Zhang L. Sun T. Woldesellassie F. Xiao X. Tao Y. (2015). Sex ratio meiotic drive as a plausible evolutionary mechanism for hybrid male sterility. *PLos Genet.* 11:e1005073. 10.1371/journal.pgen.1005073 25822261 PMC4379000

